# Drysdalin, an antagonist of nicotinic acetylcholine receptors highlights the importance of functional rather than structural conservation of amino acid residues

**DOI:** 10.1096/fba.1027

**Published:** 2019-01-10

**Authors:** Ritu Chandna, Han‐Shen Tae, Victoria A. L. Seymour, Shifali Chathrath, David J. Adams, R. Manjunatha Kini

**Affiliations:** ^1^ Protein Science Laboratory, Department of Biological Sciences National University of Singapore Singapore; ^2^ Illawarra Health and Medical Research Institute (IHMRI), University of Wollongong Wollongong NSW Australia; ^3^ Burnet Institute Melbourne VIC Australia

**Keywords:** Cys‐loop receptor recognition, nicotinic acetylcholine receptor, nonhomologous mutation, protein‐protein interaction, snake venom neurotoxin

## Abstract

Snake venom neurotoxins are potent antagonists of nicotinic acetylcholine receptors (nAChRs). Here, we describe a novel member of class 3c long‐chain neurotoxin drysdalin from the venom of *Drysdalia coronoides*. Drysdalin lacks three of the eight conserved classical functional residues critical for nAChRs interaction. Despite such a drastic alteration of the functional site, recombinant drysdalin showed irreversible postsynaptic neurotoxicity with nanomolar potency and selectively antagonizes the rodent muscle (α1)_2_β1δε, and human α7 and α9α10 nAChRs, but had no significant activity at the human α3β2, α3β4, α4β2, and α4β4 nAChRs. Substitution of Leu34 and Ala37 residues with the conserved Arg had minimal impact on the potency whereas conserved Phe replacement of residue Arg30 substantially reduced or abolished inhibitory activity. In contrast, truncation of the 24‐residue long C‐terminal tail leads to complete loss in (a) activity at α9α10 nAChR; and (b) irreversibility with reduced potency at the muscle and α7 nAChRs. Overall, the non‐conserved Arg30 residue together with the uniquely long C‐terminal tail contribute to the inhibitory activity of drysdalin at the nAChRs suggesting, at least for drysdalin, functional rather than sequence conservation plays a critical role in determining the activity of the toxin.

## 1 INTRODUCTION

Nicotinic acetylcholine receptors (nAChRs) represent a family of cation permeant pentameric ligand‐gated ion channels assembled from a pool of 17 homologous polypeptides (α1‐10, β1‐4, γ, ε, and δ)^1^ and are responsible for micro to millisecond neurotransmission.^2^ The muscle‐type nAChR is the major neurotransmitter receptor at the neuromuscular junction, whereas “neuronal” type nAChRs are widely expressed in the central and peripheral nervous systems as well as in nonneuronal cells.^3^ Neuronal nAChRs are involved in regulatory (neurotransmitters release, regulation of gene expression, and neuroprotection) as well as complex (cognition, learning and memory, arousal, reward, motor control, and analgesia) physiological functions. Apart from these physiological roles, nAChRs have been linked to many neurodegenerative and psychiatric disorders such as Alzheimer's disease (α4β2, α7),^4^ Parkinson's disease (α6β2*, α4β2*; where * signifies other subunits),^5^ schizophrenia (α7), and pain (α4β2, α7, α3*, α9α10).^6^ Thus, ligands that modulate their function are increasingly being developed for the treatment of these diseases.

Many nAChR agonists and antagonists are isolated from plants (nicotine, d‐tubocuraine, methyllycaconitine), bacteria (invermectin), algae (anatoxin‐a), and animals such as cone snails (conotoxins), corals (lophotoxin), toads (epibatidine), and snakes (α‐bungarotoxin [Bgtx]) (reviewed in^7^). Snake venom is one of the most abundant sources of natural ligands interacting with nAChRs with high specificity and potency. One such toxin isolated from *Bungarus multicinctus* is Bgtx. As with Bgtx, most snake venom‐derived nAChRs antagonists belong to the three‐finger toxin (3FTx) family with three β‐stranded “fingers” extending from a central core containing four conserved disulphide bridges. Although members of this family share a common fold, they bind to distinct receptors, enzymes, and ion channels, and exhibit a wide variety of biological effects utilizing unique functional sites (reviewed in^8^). Neurotoxins are one of the largest members of the 3FTx family that interfere with cholinergic transmission in the central and peripheral nervous systems.

Three‐finger neurotoxins can be broadly classified as curare‐mimetic or α‐neurotoxins,^9^ κ‐toxins,^10^ and Ω‐neurotoxins^11^ that antagonize nAChRs and muscarinic toxins that target muscarinic acetylcholine (ACh) receptors. The α‐neurotoxins, based on their chain length and disulphide linkages, are divided into short‐chain α‐neurotoxins (SNTXs), long‐chain α‐neurotoxins (LNTXs), and nonconventional α‐neurotoxins.^12^ SNTXs have 60‐62 amino acid (aa)^13^ residues with four conserved disulphide bonds, whereas LNTXs have 66‐74 aa residues with an additional disulphide bonds at the tip of the second loop. Both SNTXs and LNTXs bind to the muscle nAChRs (nanomolar to picomolar affinity), but only LNTXs inhibit neuronal α7, α9, and α9α10 nAChRs with high affinity (nanomolar).^14^ The functional sites of SNTX erabutoxin a, and LNTXs, α‐cobratoxin (Cbtx), and Bgtx, have been well characterized.^15^, ^16^, ^17^, ^18^ These toxins use a number of structurally equivalent residues to interact with the muscle nAChRs consisting of Lys27/Lys23, Trp29/Trp25, Asp31/Asp27, Phe32/Phe29 Arg33/Arg33, Lys47/Lys49 (erabutoxin a/Cbtx numbering). In addition to these common residues, SNTXs and LNTXs utilize specific residues for receptor‐recognition. Erabutoxin a utilizes His6, Gln7, Ser8, Ser9, and Gln10 at the tip of loop I and Tyr25, Gly34, Ile36, and Glu38 of loop II to interact with muscle nAChRs. Interestingly, Cbtx utilizes loop II residues (Trp25, Asp27, Phe29, Arg33, Arg36, and Phe65) to bind to both neuronal α7 and *Torpedo* nAChRs. In addition, it also uses receptor‐specific residues: Lys23 and Lys49 for recognition of *Torpedo* nAChR and Ala28, Cys26‐Cys30 and Lys35 for α7 nAChR.^15^, ^16^, ^17^ Taken together, neurotoxins use a common core of critical residues for binding and additional residues to determine the specificity of their molecular target.

The commonly accepted dogma that all LNTXs with a fifth disulphide block neuronal α7 nAChRs was challenged by some “classical” long‐chain α‐neurotoxins that have poor binding affinity for α7 nAChR.^19^, ^20^ The authors proposed that Arg33Leu and Phe29Arg substitutions in the “neuronal pharmacophore” region may be responsible for the lack of affinity. Thus, the substitutions in the key functional residues may lead to almost complete loss of binding affinity.

In our previous study, the venom of an Australian elapid snake *Drysdalia coronoides* was profiled to be rich in 3FTx and serine protease inhibitors.^21^ A variety of 3FTxs had sequences homologous to SNTX‐ and LNTX‐postsynaptic α‐neurotoxins, with several conserved functional residues involved in recognizing nAChRs. Among them, five 3FTx isoforms, represented by LNTX 13, 43, 173, 346, and 146R were the longest (mature protein of 87 aa residues) of all the 3FTxs. This particular group of 3FTxs have unusually long C‐terminal tail (24 aa residues). Furthermore, three functionally important residues were substituted by non‐conserved residues, namely Phe30Arg, Arg34Leu, and Arg37Ala. In the first substitution, an aromatic, hydrophobic residue is replaced by a charged, hydrophilic residue, whereas in the latter two substitutions, a positively charged, hydrophilic residue is replaced by an aliphatic, hydrophobic residue. Thus, they belong to a distinct class of LNTXs. We chose to work on the most abundant isoform, LNTX 13. Here we report the recombinant expression and purification, and pharmacological and electrophysiological characterization of drysdalin (*Drysdalia* toxin) – the first member of this novel group of 3FTxs. Interestingly, despite the substitution of functionally conserved residues in the loop II, drysdalin exhibits nanomolar postsynaptic irreversible neurotoxicity. It inhibits adult rodent muscle α1β1δε and human neuronal α7 and α9α10 nAChRs with nanomolar potency. Furthermore, we describe the roles of the C‐terminal tail and the three non‐conserved residues of drysdalin in its interaction with muscle and neuronal nAChRs.

## 2 MATERIALS AND METHODS

### 2.1 Recombinant protein expression, purification, and refolding

Drysdalin and its mutants were cloned into a pET‐32a modified vector and expressed as inclusion bodies (IBs) in *Escherichia coli *SHuffle^®^ cells. The IBs were solubilized in denaturing buffer (50 mmol/L Tris‐HCl, 150 mmol/L NaCl, 6 mol/L guanidine hydrochloride, pH 8.0) and reduced with 100 mmol/L dithiothreitol (DTT) before purification on a Jupiter C18 column (4.5 × 2.1 mm). Purified proteins were refolded by dilution into the refolding buffer (50 mmol/L Tris‐HCl, 1 mmol/L ethylenediaminetetraacetic acid (EDTA), 0.5 mol/L guanidine hydrochloride, 20% glycerol, 1 mmol/L reduced glutathione, 1 mmol/L oxidized glutathione, pH 8.0) and purified to homogeneity on a Jupiter C18 column. The molecular mass and homogeneity of all proteins were evaluated by electrospray ionization mass spectrometry (ESI‐MS) using a LCQ Fleet^TM^ Ion Trap Mass Spectrometer (Thermo Scientific, Waltham, MA, USA). The secondary structure of all the refolded proteins were evaluated by circular dichroism (CD).

### 2.2 Ex vivo CBCM organ bath

All experiments were conducted according to the Protocol (103/08A) approved by the National University of Singapore Institutional Animal Care and Use Committee. Isolated chick biventer cervicis muscle (CBCM) experiments were conducted in a 6 ml‐organ bath chamber, containing Krebs‐Henseleit buffer bubbled with carbogen (5% CO_2 _in O_2_) at 37°C, as described previously.^22^ Neuromuscular blockade by drysdalin is expressed as percentage of the twitch height in the absence of drysdalin to the twitch height 30 minutes post exposure to drysdalin. The half‐maximal inhibitory concentration (IC_50_) was determined from concentration‐response curve fitted to a nonlinear regression function and reported with error of the fit. To test for reversibility, recovery from complete neuromuscular blockade by drysdalin was assessed by washing the tissue with Krebs solution over a 120‐minute period after 80% blockade of the twitch responses.

### 2.3 Electrophysiology

RNA preparation, oocyte preparation, and expression of nAChRs were performed as described previously.^23^ All protocols were approved by the University of Sydney Animal Ethics Committee. Briefly, stage V‐VI oocytes were obtained from *Xenopus laevis*, and defolliculated with 1.5 mg/mL Type II collagenase in OR‐2 solution. To conduct the two‐electrode voltage clamp (TEVC) experiments on the oocytes, nAChRs were expressed by microinjecting the cRNA of various receptor subunits into oocytes. Electrophysiological recordings were carried out 2‐7 days after microinjection. Oocytes expressing nAChRs were voltage clamped at a holding potential −80 ,mV.^24^ nAChR‐mediated currents were evoked by applying ACh for 1.5 seconds at a rate of 2 mL/min, at a half‐maximal effective concentration (EC_50_) of each subtype followed by washouts of 180 seconds between ACh applications. Oocytes were incubated with the toxin for 5 minutes before ACh was co‐applied. Peak ACh‐evoked current amplitude before and after toxin incubation was recorded using pClamp 9 software and measured using Clampfit 10.7 (Molecular Devices, Sunnyvale, CA, USA). Concentration‐response curves were used to determine the IC_50_ of the toxin at each receptor.

## 3 RESULTS AND DISCUSSION

### 3.1 Classification of LNTXs

Curare‐mimetic α‐neurotoxins have been classified by Endo and Tamiya into SNTXs and LNTXs.^12^ Both SNTXs and LNTXs share a similar three‐dimensional structure of 3FTxs that is held together by four conserved disulphide bonds. LNTXs have an additional fifth disulphide bond in their second loop. Although both SNTXs and LNTXs bind to the muscle nAChR subtype (picomolar), only LNTXs inhibit neuronal α7 nAChRs with high affinity (nanomolar).

**Figure 1:**
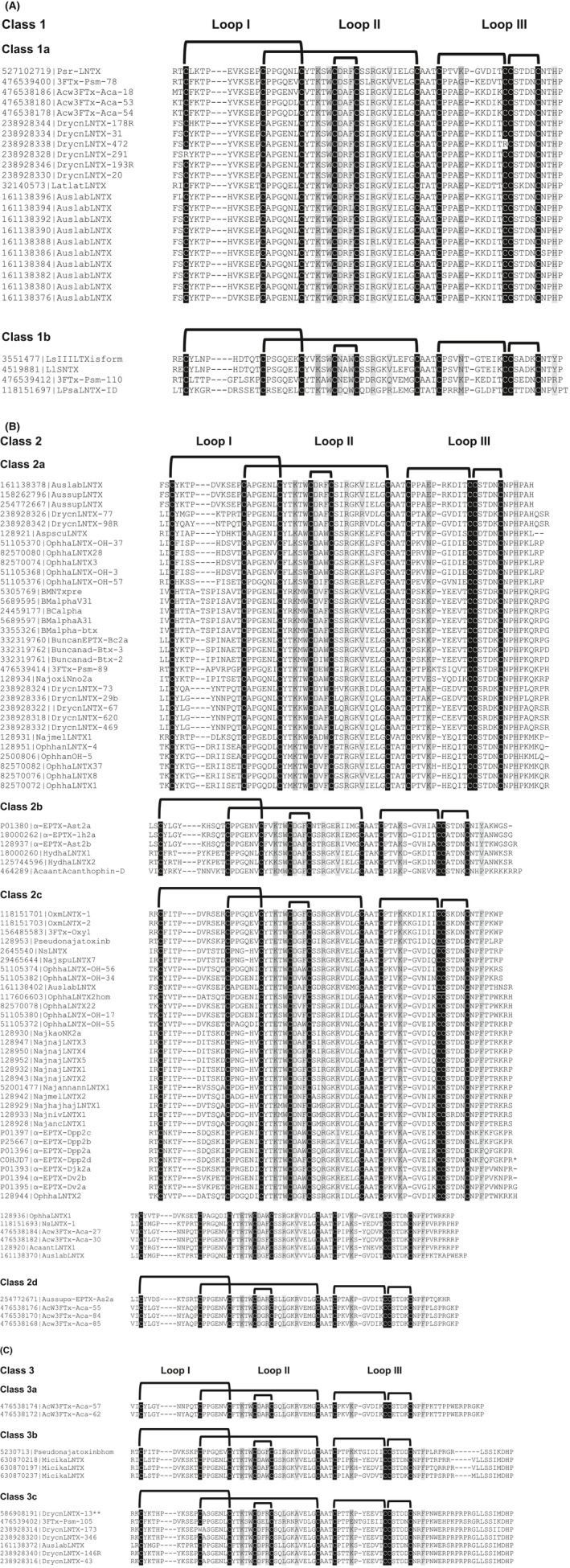
Classification of long‐chain neurotoxins (LNTXs). Primary sequence alignment of A, Class 1, B, Class 2, and C, Class 3 LNTXs listed with their accession numbers and names. The five conserved disulphide bridges (bold black lines) are formed between the cysteine residues (black columns). Light and dark gray columns, in the loops II and III, are conserved residues involved in interaction with neuronal and muscle nAChRs, respectively. The class 3c drysdalin labelled as DrycnLNTX 13 is marked by **

**Figure 2:**
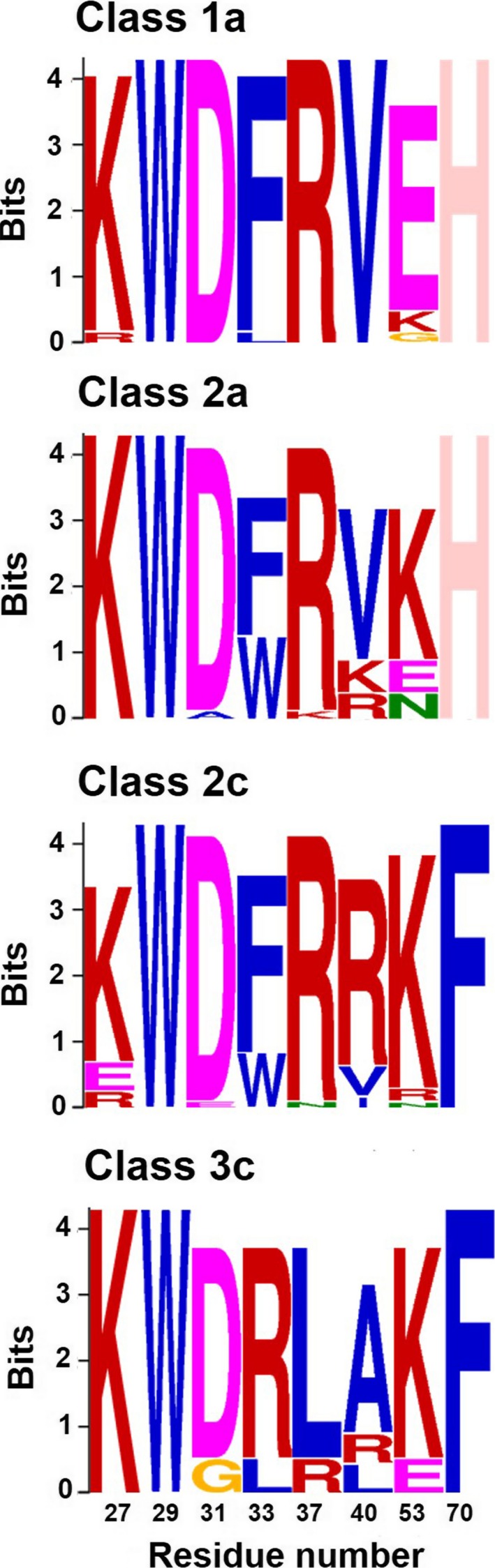
Conserved motif analysis of long‐chain neurotoxins (LNTXs). Multiple EM for Motif Elucidation (MEME) methodology used for LNTX classes 1a, 2a, 2c, and 3c representing the frequency of the conserved residues among the members of the class. The numbers on the X‐axis indicate the positions of the residue in the protein sequence. MEME diagram of classes with greater than seven toxins are represented here

Using systematic site‐directed mutagenesis of 29 residues, Antil et  al explored the role of all Cbtx three loops and identified common residues (Trp25, Asp27, Phe29, Arg33, Arg36, and Phe65) that interact with both *Torpedo *and α7 nAChRs and receptor subtype‐selective residues (Lys23 and Lys49 at *Torpedo* nAChR and Ala28, Lys35 and the Cys26‐Cys30 disulphide bond at α7 nAChR).^15^, ^16^ In the last two decades, a significant number of LNTXs have been discovered with minimal characterization of residues at the functional site. Our preliminary evaluation indicated that some of these LNTXs have distinct structural differences and therefore, we classified them further into three classes based on the number of C‐terminal tail residues after the last Cys residue in the aa sequence and variations in the functionally important residues (Figures 1 & 2).

#### 3.1.1 Class 1

Toxins with the shortest chain length (66‐68 aa residues) and only 4‐5 residues in the C‐terminus were classified into Class 1 (Figure 1A). They were further categorized as 1a and 1b based on the differences in the functionally important residues in loop II, III, and C‐terminus. We observed that two functionally important residues were substituted by non‐conserved residues, namely, Lys53 to Glu and Phe70 to His. Lys53Glu substitution reduced Cbtx affinity for both binding sites at the *Torpedo* nAChR, with one site greatly reduced by 52‐fold whereas the other by only threefold.^16^ This substitution also led to threefold reduction in affinity for the *Gallus* α7/5‐HT_3_ chimeric receptor.^15^ The impact of Phe70His substitution on the affinity is unclear as Cbtx and Bgtx have Phe and His at the homologous position, respectively. The functional impact of augmenting the short C‐terminus has been studied in both Cbtx and Bgtx. A threefold reduction in the affinity for the α7/5‐HT_3_ nAChR chimera was reported for the Cbtx C‐terminal deletion mutant Cbtx[ΔPro66‐Pro71]^15^ whereas, truncation of Bgtx seven C‐terminal residues (Bgtx[ΔHis68‐Gly74]) decreased the apparent binding affinity to the *Torpedo* nAChR by sevenfold.^18^

#### 3.1.2 Class 2

Toxins with intermediate chain length (68‐75 aa residues) and 6‐13 C‐terminal residues were classified into Class 2 (Figure 1B). They were further categorized into four subclasses (2a‐d) based on the differences in the functionally important residues in loop II, III, and C‐terminus. Class 2a toxins share a common His70 residue with 6‐9 residues in the C‐terminus, whereas Class 2c toxins have a conserved Phe residue at position 70 with slightly longer C‐terminal tail consisting of 7‐13 residues (Figure 2). Class 2b toxins have either Tyr/Val/Pro residue at position 70. Class 2d toxins share common Arg33 and Leu37 residues, whereas toxins in subclasses 2a‐c have an aromatic residue and a positively charged Arg residue at position 33, 37, respectively. The key feature of most toxins in groups 2a and 2c is the presence of two positively charged Lys and Arg residues at the C‐terminus. Removal of the corresponding Lys70 and Arg72 residues in Bgtx[ΔHis68‐Gly74] resulted in a sevenfold reduction in the binding activity at the *Torpedo* nAChR.^18^

#### 3.1.3 Class 3

Toxins with the longest chain length (79‐88 aa residues) and the longest C‐terminus (17‐24 aa residues) were classified into Class 3 (Figure 1C). They were further divided into three subclasses (3a‐c) based on the length and sequence identity of the C‐terminus. Class 3a toxins have the shortest tail among Class 3 toxins with only 17 aa residues in the C‐terminal tail. Classes 3b and 3c share similar C‐terminal sequences, however, LNTXs of class 3c have additional 4‐6 residues (mostly Pro and Arg residues) upstream of the homologous C‐terminal region. For majority of class 3c toxins, key functionally important residues Phe/Trp33 and Arg37, and Arg40 are substituted with Arg/Leu and Ala/Leu, respectively (Figure 2). Drysdalin is grouped in class 3c α‐neurotoxin (labelled DrycnLNTX‐13 in Figure 1C) with a long 24‐aa C‐terminal tail and the consensus LNTX residues Phe30, Arg34, and Arg37 are substituted by residues Arg, Leu and Ala, respectively. The importance of these residues positions has been explained above. Interestingly, the length of class 3c toxins are similar to those of nAChR interacting human Ly6 proteins (Lynx1, PATE4, SLURP1, and SLURP2),^25^ but they share poor sequence similarity (19%‐25%) and class 3c toxins have much longer C‐terminal tail segments.

To date, the function of only Cbtx (Class 2c) and Bgtx (Class 2a) has been well characterized. The impact of several key functional residue substitutions and changes in the C‐terminal segments on the nAChR subtype selectivity of class 3c members are unknown. Here, we characterize the first member of class 3c α‐neurotoxin, drysdalin (DrycnLNTX‐13). In this toxin, three out of the eight functional residues (37.5%) are replaced by non‐conserved residues, and additionally it has a 24 aa residue‐long C‐terminal tail. Here, we describe the function of drysdalin and the role of these non‐conserved residues and C‐terminal tail in the interactions with various nAChRs.

### 3.2 Recombinant expression and purification of drysdalin

**Figure 3:**
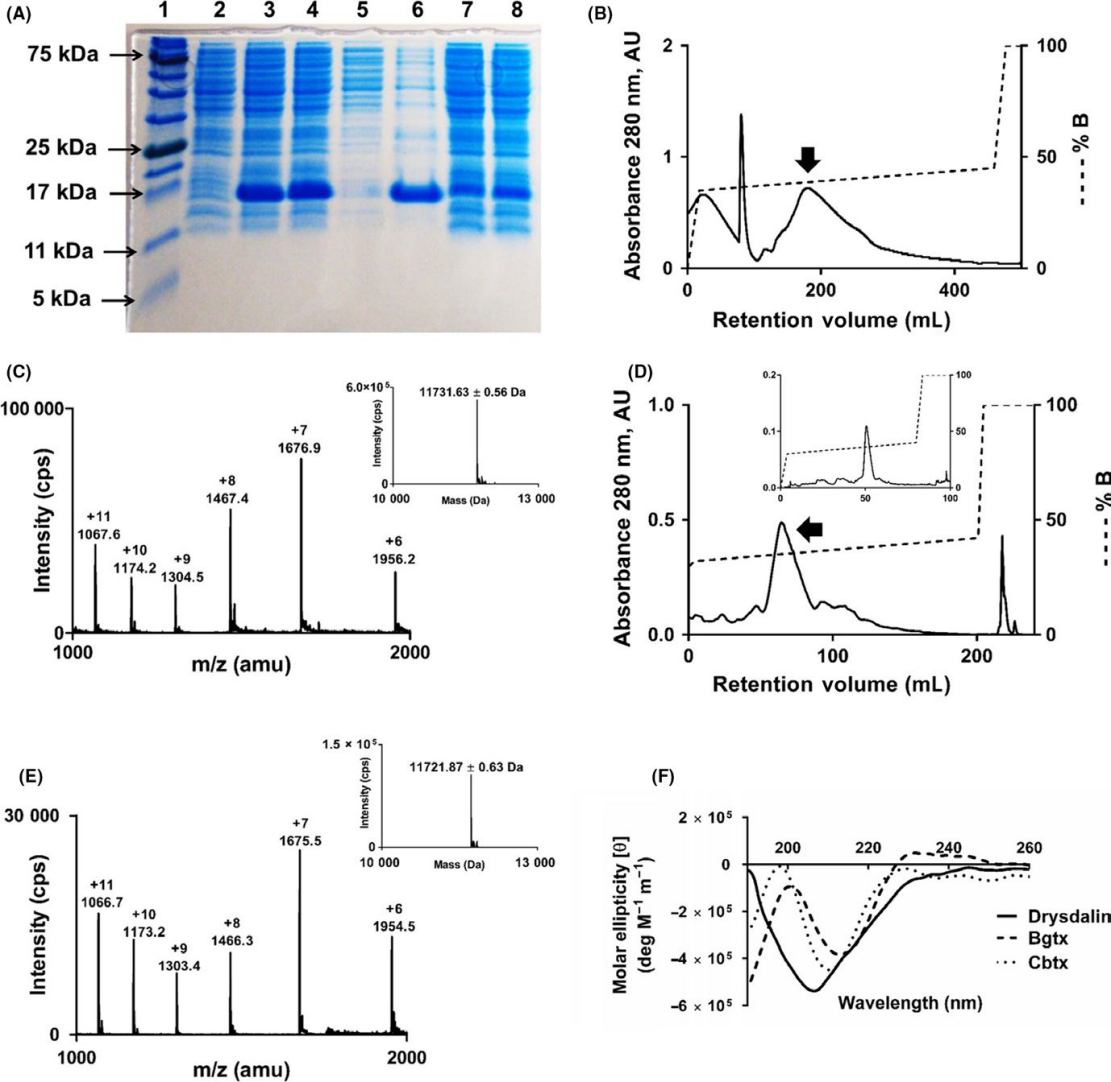
Recombinant expression, purification, and secondary structure of drysdalin. A, sodium dodecyl sulfate–polyacrylamide gel electrophoresis (SDS‐PAGE) analysis of recombinant drysdalin expression. Lane 1: protein ladder; lane 2: whole cell lysate before protein induction; lanes 3 and 4: whole cell lysates after 0.5 and 1.0 mmol/L isopropyl β‐D‐1‐thiogalactopyranoside (IPTG) induction (I) at 37°C for 4 hours, respectively; lane 5: soluble fraction; lane 6: inclusion bodies (IBs); lane 7 and 8: cell pellet washes (W1 and W2, respectively) with 1% Triton X‐100 buffer. B, high‐performance liquid chromatography (HPLC) profile of reduced drysdalin purified from inclusion bodies on a Jupiter C18 (5 μ, 300 Å, 4.5 × 21.2 mm) preparative column. Drysdalin was eluted from the column at a flow rate of 5 mL/min with gradient of 35%‐45% buffer B (80% Acetonitrile [ACN] in 0.1% trifluoroacetic acid [TFA]) over five column volumes (CV). Arrow indicates the fraction containing drysdalin. C, electrospray ionization mass spectrometry (ESI‐MS) profile of the purified and reduced drysdalin. The spectrum shows a series of multiply charged ions, corresponding to a single, homogenous protein with a molecular mass of 11731.63 ± 0.56 Da. *Inset*, reconstructed mass spectrum of reduced drysdalin. D, HPLC profile of refolded drysdalin from the refolding buffer on a Jupiter C18 (5 μ, 300 Å, 4.5 × 10 mm) semi‐preparative column. Refolded drysdalin was eluted from the column at a flow rate of 2 mL/min with a gradient of 32%‐42% buffer B over 10 CV. The arrow indicates fraction containing major conformer of the refolded drysdalin. *Inset*, repurification of the major conformer of drysdalin on Jupiter C18 (5 μ, 300 Å, 4.5 × 4.6 mm) analytical column. Refolded drysdalin was eluted from the column at a flow rate of 1 mL/min with gradient of 32%‐42% buffer B over 20 CV. E, ESI‐MS profile of the reverse phase‐high performance liquid chromatography (RP‐HPLC) fraction containing the refolded drysdalin. The spectrum shows a series of multiply charged ions, corresponding to a single, homogenous protein with a molecular mass of 11721.87 ± 0.63 Da. *Inset*, reconstructed mass spectrum of refolded drysdalin. F, Far UV‐spectrum of refolded drysdalin shows a minimum around 206‐209 nm indicating β‐sheeted secondary structure in the protein. Typical LNTXs, α‐bungarotoxin (Bgtx), and α‐cobratoxin (Cbtx) are shown for comparison

The venom yield of *D. coronoides* is typically 2‐3 mg per milking (Mr Peter Mirtschin, Venom Supplies Pty Ltd., Tanunda, South Australia; unpublished observations). Due to the low venom yield, we recombinantly expressed drysdalin to obtain large quantities of the protein. Drysdalin was expressed predominantly as IBs (Figure 3A). The Triton X‐100‐washed IBs were solubilized in a denaturing buffer, reduced with DTT, and purified to homogeneity by reverse phase‐high performance liquid chromatography (RP‐HPLC) (Figure 3B). ESI‐MS data of reduced drysdalin showed six peaks of mass/charge (m/z) ratios ranging from +6 to +11 (Figure 3C). The reconstructed mass spectrum showed a molecular mass of 11731.63 ± 0.56 Da (Figure 3C inset), matching the calculated 11732.2 Da mass. After refolding, all the conformers were separated on a Jupiter semi‐preparative C18 column (Figure 3D) and the major peak was rechromatographed on an analytical C18 column to homogeneity (Figure 3D inset). ESI‐MS data of refolded drysdalin showed six peaks of mass/charge (m/z) ratios ranging from +6 to +11 (Figure 3E). The reconstructed mass spectrum of the refolded drysdalin showed a molecular mass of 11721.87 ± 0.63 Da (Figure 3E inset), indicating a loss of ten protons due to the formation of five disulphide bonds. The CD spectrum of drysdalin showed a minimum at 206‐209 nm, indicating the presence of β‐sheeted secondary structure characteristic of 3FTxs (Figure 3F).

### 3.3 Drysdalin is a potent postsynaptic neurotoxin

To examine the biological effects of drysdalin, the refolded recombinant drysdalin was injected intraperitoneally into adult mice at a dose of 0.25 and 3 mg/kg of body weight. Both mice showed typical signs of peripheral neurotoxicity such as hind limb paralysis, laboured breathing, and finally death (after 46 minutes at 3 mg/kg) presumably due to respiratory paralysis.^26^, ^27^ No gross changes were observed in the internal organs of the mice.

**Figure 4:**
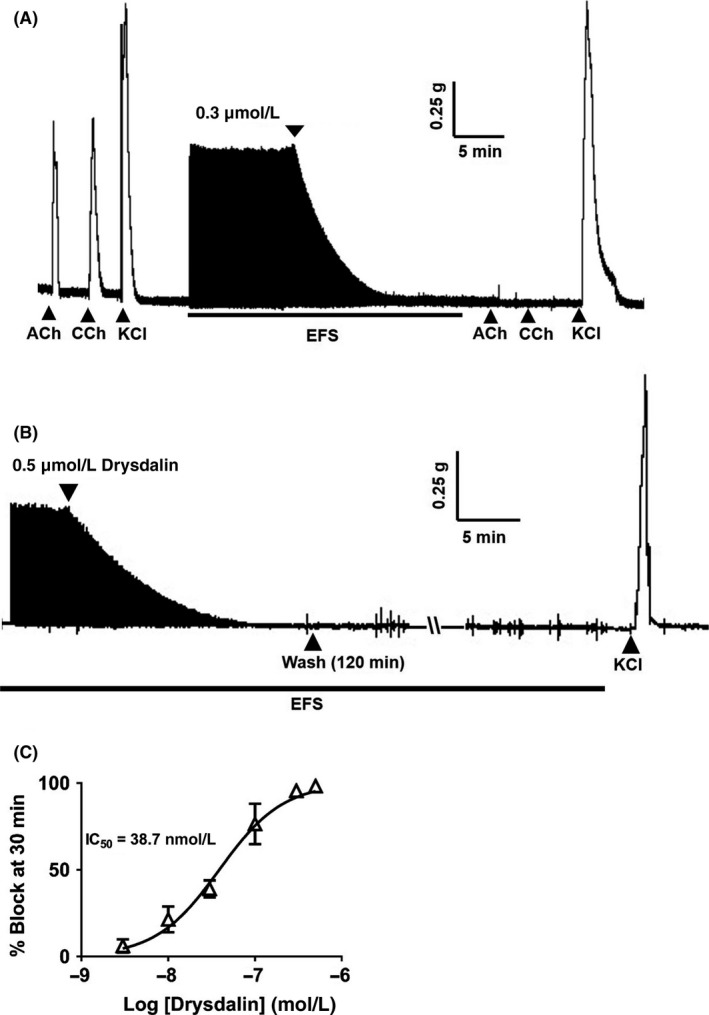
Postsynaptic neurotoxicity of drysdalin on chick biventer cervicis muscle (CBCM) preparation. A, Representative traces showing the effect of drysdalin (0.3 μmol/L) on the twitch response of CBCM. Twitch response of the muscle was evoked by electrical field stimulation (EFS) (indicated by the black bar) and by agonists acetylcholine (ACh 300 μmol/L), CCh (10 μmol/L), and potassium chloride (KCl 30 mmol/L). Upward pointing arrows indicate point of agonist application. B, Irreversibile block of the nerve‐evoked twitch response by drysdalin in CBCM. A segment of trace shows the irreversible block of nerve‐evoked twitch responses by drysdalin (0.5 μmol/L) in CBCM after 120 minutes wash. Contractions induced by KCl (30 mmol/L) after drysdalin block indicate the viability of the muscle. Arrows indicate point of application. C, Concentration‐dependent inhibition of CBCM contractile responses 30 minutes post exposure to drysdalin. Each data point is the mean ± SEM of two to four experiments

The mode of action of drysdalin's neurotoxic effects was characterized in CBCM preparations. Drysdalin irreversibly blocked postsynaptic neuromuscular indirect (nerve) electrical field stimulation (EFS)‐evoked twitch responses without direct myotoxicity as indicated by the absence of contractile responses to agonists such as ACh (300 µmol/L) and carbachol (CCh, 10 µmol/L), whereas the response to KCl (30 mmol/L), which causes direct muscle stimulation, was not affected (Figure 4A, B). Drysdalin showed a concentration‐dependent postsynaptic effect on CBCM giving an IC_50_ of 38.7 ± 5.7 nmol/L (n = 3) (Figure 4C). In comparison to Bgtx (IC_50_ on CBCM = 12.1 ± 5.4 nmol/L) (n ≥ 3),^28^ drysdalin was about threefold less potent. Thus, despite the absence of the three functionally conserved residues of α‐neurotoxins, drysdalin exhibited almost irreversible and potent (nanomolar) postsynaptic neuromuscular block.

### 3.4 Drysdalin inhibits heterologously expressed rodent and human nAChRs

**Figure 5:**
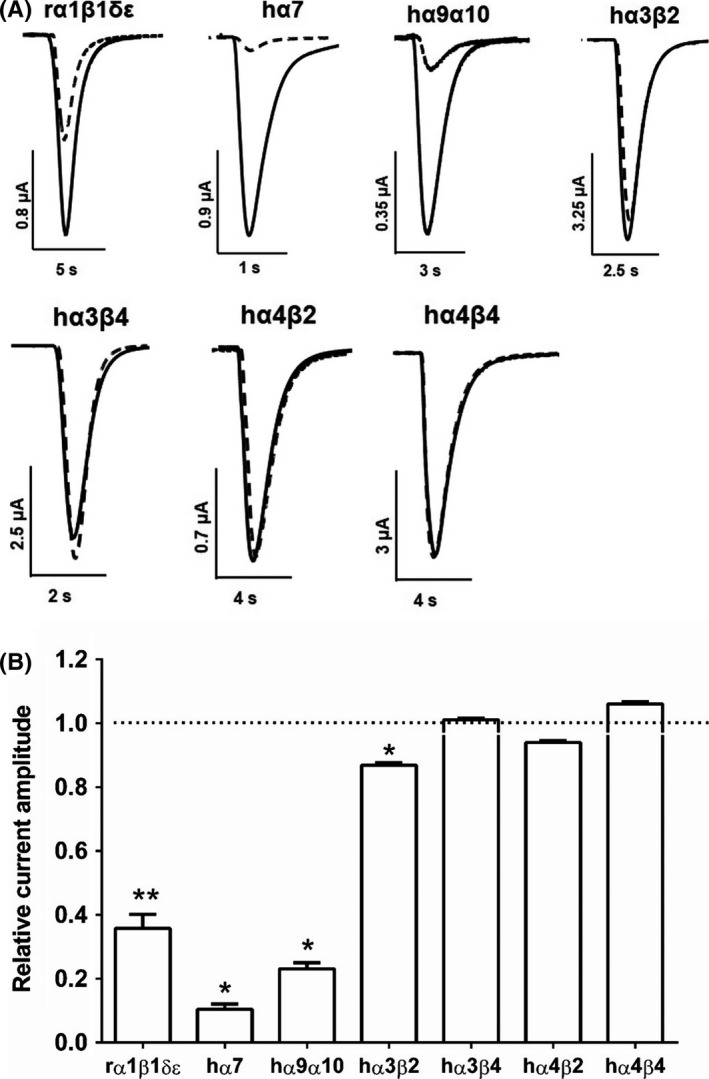
Drysdalin inhibition of nAChRs subtypes expressed in *Xenopus* oocytes. A, Superimposed representative ACh‐evoked currents recorded from *Xenopus* oocytes expressing rα1β1δε, hα7, hα9α10, hα3β2, hα3β4, hα4β2, and hα4β4 nAChRs in the absence (solid line) and presence of 30 nmol/L drysdalin (dashed line). B, Bar graph of drysdalin (30 nmol/L) inhibition of ACh‐evoked peak current amplitude mediated by rα1β1δε, hα7, hα9α10, hα3β2, hα3β4, hα4β2, and hα4β4 nAChRs. Whole‐cell currents mediated by hα3β2 and hα4β2 were activated by 6 µmol/L ACh; hα3β4, hα4β4, hα7, and rα1β1δε nAChRs were activated by 300, 3, 200 and 1 µmol/L ACh, respectively (mean ± SEM, n = 3‐5) (unpaired Student *t* test; * *P* < 0.0001, ** *P* = 0.0001 vs relative current amplitude of 1).

As LNTXs bind to both muscle and neuronal α7 nAChRs,^12^ we tested the activity of drysdalin on nAChRs heterologously expressed in *X. laevis* oocytes. Drysdalin selectively blocked the rodent (r) muscle‐type α1β1δε and human (h) neuronal α7 and α9α10 subtypes, and had no significant inhibitory activity at hα3β2, hα3β4, hα4β2, and hα4β4 nAChRs when tested up to 30 nmol/L (Figure 5).

**Figure 6:**
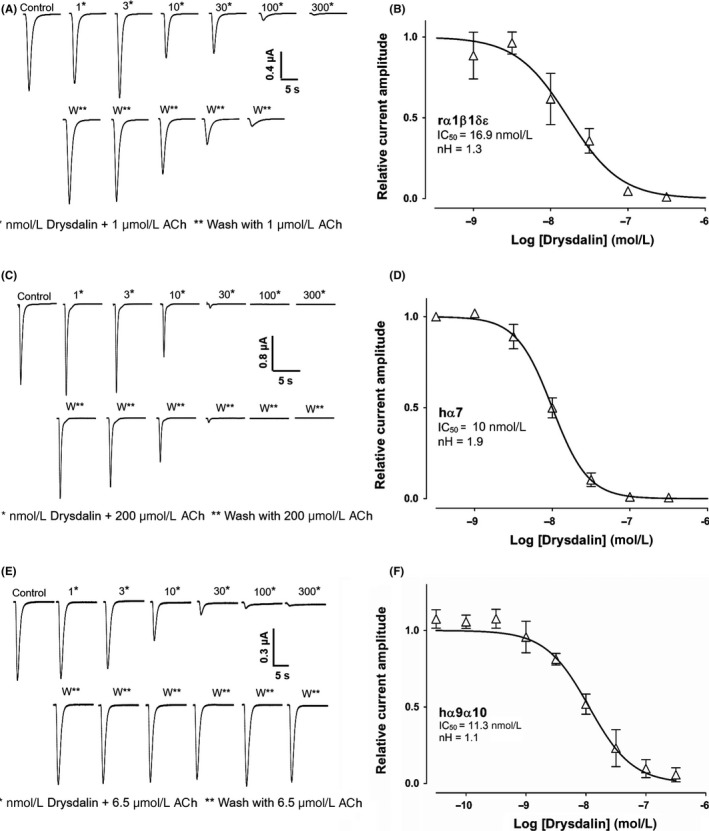
Drysdalin inhibition of rα1β1δε, hα7, and hα9α10 nAChRs subtypes. A, Representative ACh (1 μmol/L)‐evoked currents recorded from *Xenopus* oocytes expressing rodent muscle α1β1δε nAChRs in the absence (control) and presence of 1‐300 nmol/L drysdalin (top row), and following washout between applications of drysdalin (bottom row). B, Concentration‐response curve of relative ACh‐evoked current amplitude mediated by rα1β1δε nAChRs in the presence of drysdalin gave an IC_50_ of 16.9 ± 3.6 nmol/L (n = 3) and Hill coefficient (n^H^) of 1.3. C, Representative ACh (200 μmol/L)‐evoked currents recorded from oocytes expressing human (h)α7 nAChR in the absence (control) and presence of 1‐300 nmol/L drysdalin (top row), and following washout between applications of drysdalin (bottom row). D, Concentration‐response curve of relative ACh‐evoked current amplitude mediated by hα7 nAChR in the presence of drysdalin gave an IC_50_ of 9.98 ± 0.6 nmol/L (n = 5) and n^H^ of 1.9. E, Representative ACh (6.5 μmol/L)‐evoked currents recorded from oocytes expressing hα9α10 nAChRs in the absence (control) and presence of 1‐300 nmol/L drysdalin (top row), and following washout between applications of drysdalin (bottom row). F, Concentration‐response curve of relative ACh‐evoked current amplitude mediated by hα9α10 nAChRs in the presence of drysdalin gave an IC_50 _of 11.3 ± 0.2 nmol/L (n = 6‐9) and n^H^ of 1.1

Drysdalin inhibited ACh‐evoked currents mediated by rα1β1δε, hα7, and hα9α10 nAChRs in a concentration‐dependent manner **(**Figure 6). Drysdalin irreversibly inhibited the rα1β1δε nAChR subtype (IC_50_ = 16.9 ± 3.6 nmol/L), with a Hill coefficient of 1.3 indicating similar affinities to the two ACh binding sites (α1/δ, α1/ε)^29^ (Figure 6A‐B). In comparison to drysdalin, the two other LNTXs, Bgtx and Cbtx, also irreversibly inhibited the rα1β1δε nAChRs with similar (IC_50_ = 14.0 ± 1.7 nmol/L) and 13‐fold higher potency (1.3 ± 0.1 nmol/L), respectively (Table 1).

**Table 1:** IC_50_ values of drysdalin, α‐bungarotoxin (Bgtx) and α‐cobratoxin (Cbtx) at rodent (r)α1β1δε, human (h)α7, and hα9α10 nAChRs expressed in *Xenopus* oocytes

Antagonist	rα1β1δε			hα7			hα9α10		
	IC_50 _(nmol/L)	n^H^	n	IC_50_ (nmol/L)	n^H^	n	IC_50_ (nmol/L)	n^H^	n
Drysdalin	16.9 ± 3.6	1.3	3	10.0 ± 0.6	1.9	5	11.3 ± 0.2	1.1	6‐9
Bgtx	14.0 ± 1.7	1.8	3	5.6 ± 0.7	1.4	7	9.7 ± 0.7	1.1	5‐6
Cbtx	1.3 ± 0.1	1.9	4	2.8 ± 0.1	2.2	5	7.9 ± 0.7	0.7	4‐6

IC_50_ values indicated are with their respective error of the fit.

At hα7 nAChR, drysdalin potently inhibited ACh‐evoked currents with an IC_50_ of 10.0 ± 0.6 nmol/L (Figure 6D), and a Hill coefficient of 1.9. Both Bgtx and Cbtx were more potent inhibitors of hα7 nAChR compared to drysdalin with IC_50_ of 5.6 ± 0.7 nmol/L (twofold) and 2.8 ± 0.1 nmol/L (fourfold), respectively (Table 1). All three LNTXs, drysdalin (Figure 6C), Bgtx, and Cbtx irreversibly inhibited hα7 nAChRs.

At hα9α10 nAChR, drysdalin concentration‐dependently and reversibly inhibited ACh‐evoked currents with an IC_50_ of 11.3 ± 0.2 nmol/L (Figure 6F). Bgtx and Cbtx also inhibited hα9α10 nAChRs in a concentration‐dependent manner with an IC_50 _of 9.7 ± 0.7 nmol/L and 7.9 ± 0.7 nmol/L, respectively (Table 1), and most importantly, the inhibition of hα9α10 nAChR was reversible for both LNTXs. The reversibility of drysdalin inhibition of hα9α10 contrasts with the non‐reversible inhibition at rα1β1δε and hα7 nAChRs which may arise from differences in the binding site residues at the subunit interfaces (Supporting Information Figure S5).

### 3.5 C‐terminal tail plays a critical role in the activity of drysdalin

Residues in the C‐terminus of Bgtx and Cbtx have been implicated in interactions with the nAChRs. Both Bgtx[ΔHis68‐Gly74]^18^ and Cbtx[ΔPro66‐Pro71]^15^ C‐terminus deleted analogues had reduced apparent affinity at the muscle‐type *Torpedo *nAChR (sevenfold) and *Gallus* α7/5‐HT_3_ chimeric receptor (threefold), respectively. Additionally, alanine substitution of the Cbtx C‐terminal residues resulted in reduced affinities at the aforementioned receptors. At the *Torpedo *nAChR, Cbtx[Phe65Ala] had sevenfold affinity loss^16^ whereas at the α7/5‐HT_3 _chimera, the affinities of both Cbtx[Phe65Ala] and Cbtx[Pro66Ala] were reduced by 16‐ and threefold, respectively.^15^ Drysdalin has an extended 24 residue‐long C‐terminal tail compared to other LNTXs (Figure 1C). To determine the contribution of the C‐terminal tail in interactions with nAChRs, truncated drysdalin (tDrysdalin) lacking 20 aa residues of the C‐terminus was designed and recombinantly expressed. (Supporting Information Figures S2, S3D). The refolded truncated protein showed mass corresponding to the calculated value (Supporting Information Table S1) and the overall structure of tDrysdalin was similar to the full‐length protein (Supporting Information Figure S4).

**Figure 7:**
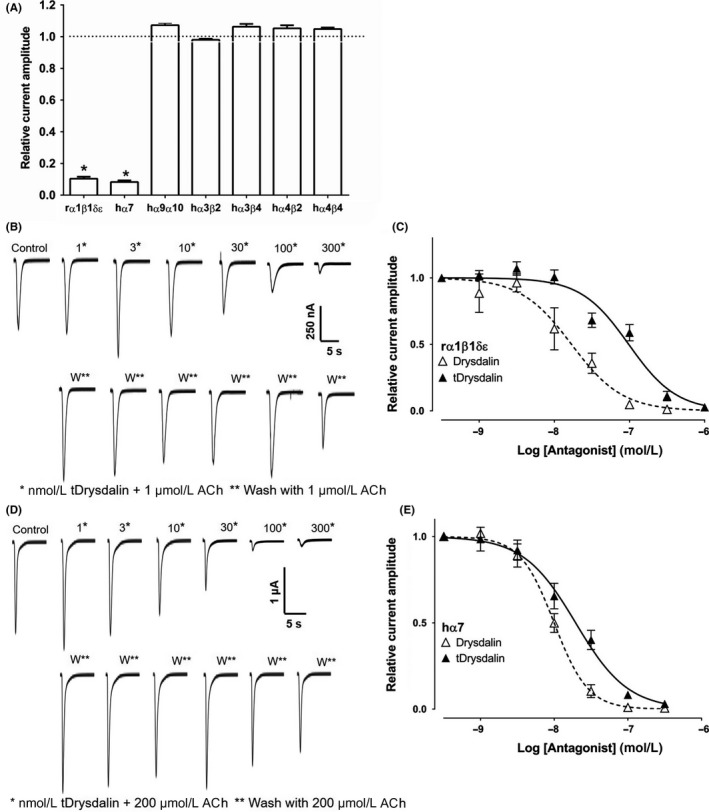
tDrysdalin inhibition of nAChRs subtypes. A, Bar graph of tDrysdalin (100 nmol/L) inhibition of ACh‐evoked peak current amplitude mediated by rα1β1δε, hα7, hα9α10, hα3β2, hα3β4, hα4β2, and hα4β4 nAChRs. Whole‐cell currents at hα3β2 and hα4β2 were activated by 6 µmol/L ACh, hα3β4, hα4β4, hα7, and rα1β1δεnAChRs were activated by 300, 3, 200, and 1 µmol/L ACh, respectively (mean ± SEM, n = 3‐6) (unpaired Student t test; *P < 0.0001 vs relative current amplitude of 1). B, Representative ACh (1 μmol/L)‐evoked currents recorded from *Xenopus* oocytes expressing rodent muscle α1β1δε nAChRs in the absence (control) and presence of 1‐300 nmol/L tDrysdalin (top row), and following washout between applications of tDrysdalin (bottom row). C, Concentration‐response curve of the relative ACh‐evoked current amplitude mediated by rα1β1δε nAChRs in the presence of tDrysdalin gave an IC_50_ of 97.4 ± 14 nmol/L (n = 3) and 1.3 as Hill coefficient (n^H^). D, Representative ACh (200 μmol/L)‐evoked currents recorded from oocytes expressing human(h)α7 nAChR in the absence (control) and presence of 1‐300 nmol/L tDrysdalin (top row), and following washout between applications of tDrysdalin (bottom row). E, Concentration‐response curve of the relative ACh‐evoked current amplitude mediated by hα7 nAChRs in the presence of tDrysdalin gave an IC_50_ of 19.5 ± 2.1 nmol/L (n = 3) and n^H^ of 1.3

tDrysdalin retained the selectivity of drysdalin to inhibit the muscle‐type rα1β1δε‐ and neuronal hα7‐mediated ACh‐evoked currents (Figure 7A) although at a lower 2‐5 fold potency (IC_50_ = 97.6 ± 14.0 nmol/L and 19.5 ± 2.1 nmol/L, respectively [Figure 7C, E, Table 2]). However, unlike drysdalin, 100 nmol/L tDrysdalin did not inhibit ACh‐evoked currents mediated by hα9α10 (Figure 7A), indicating a role for the C‐terminal tail in recognizing this nAChR subtype. Furthermore, tDrysdalin inhibition of both rα1β1δε and hα7 nAChRs was reversible as observed from the recovery of ACh‐evoked current amplitude after 3 minutes washout (Figure 7B, D).

**Table 2:** IC_50_ values of drysdalin and tDrysdalin at rodent (r), and human (h) nAChRs expressed in *Xenopus* oocytes

nAChR subtypes	Drysdalin	tDrysdalin
	IC_50 _(nmol/L)	IC_50 _(nmol/L)
rα1β1δε	16.9 ± 3.6 Irreversible	97.6 ± 14.0 Reversible
hα7	10.0 ± 0.6 Irreversible	19.5 ± 2.1 Reversible
hα9α10	11.3 ± 0.2 Reversible	Inactive^b^
hα3β2	13% inhibition^a^ Reversible	Inactive^b^
hα3β4	Inactive^a^	Inactive^b^
hα4β4	Inactive^a^	Inactive^b^
hα4β2	Inactive^a^	Inactive^b^

IC_50_ values indicated are with their respective error of the fit (n = 3‐9)

a Concentration of drysdalin tested is 30 nmol/L.

b Concentration of tDrysdalin tested is 100 nmol/L.

The effect of Bgtx and Cbtx truncation on the reversibility of ACh current inhibition is currently unknown. Here, we report for the first time the effect of C‐terminal residue removal on the reversibility of any three‐finger α‐neurotoxin on the inhibition of ACh‐evoked currents mediated by nAChRs.

### 3.6 “Reverting” mutations of non‐conserved functional residues has no significant effect on the activity of drysdalin

Structural studies of LNTXs strongly suggest the involvement of three conserved residues (one Phe and two Arg) in binding of the toxins to their molecular targets. The interaction of these residues was observed in the crystal structure of *Lymnaea stagnalis *(*Ls*)‐acetylcholine binding protein (AChBP) with Cbtx. *Ls*‐AChBP principal subunit residues Tyr185 and Tyr192 form π‐π interactions with Cbtx Phe29 and additionally Tyr185 interacts with Cbtx Arg33 (1st Arg) and Arg36 (2nd Arg) via cation‐π interactions (Supporting Information Figure S6A).^30^ Similar cation‐π interactions are also energetically favourable in the binding of Cbtx to an 18‐mer cognate peptide derived from the *Torpedo* muscle nAChR (Supporting Information Figure S6B).^31^

An elaborate cation‐π interaction system is also involved in the interaction between Bgtx loop II residues Phe32 and Arg36 (1st Arg) with residues Trp93, Tyr190, and Tyr198 of the monomeric mouse α1 nAChR extracellular domain (Supporting Information Figure S6C).^32^ At the α7/Ls‐AChBP chimera, Bgtx loop‐II residues Arg36 and Phe32 form a π‐cation stack that aligns edge‐to‐face with the conserved α7‐loop C Tyr184 and additionally, Arg36 interacts with receptor residues Tyr91, Trp145 and Tyr191 (Supporting Information Figure S6D).^33^ Mutational studies show that Tyr184 alone is required for the high affinity of Bgtx to α7 receptor as seen from the complete loss in the affinity for the mutation Tyr184Thr. Single residue mutations Tyr184Phe and Tyr191Thr do not affect Bgtx apparent affinity at the receptor. However, with both mutations the apparent affinity decreases by sixfold.^34^ Hence, conserved residues Phe and Arg of Cbtx and Bgtx form an extensive cation‐π interaction system in the aromatic ligand‐binding pocket of muscle as well as neuronal nAChRs. However, there are subtle differences in the interactions of LNTXs with different subtypes of nAChRs.

Structure‐function relationship studies of LNTXs also indicate the importance of Phe and Arg residues in recognizing the muscle and neuronal nAChRs. Cbtx loop II mutants [Phe29Leu/Ala], [Arg33Glu], and [Arg36Ala] had decreased binding to the *Torpedo* nAChR and α7/5‐HT_3_ receptor.^15^, ^16^ In contrast, substitution of Phe29 with conserved Trp residue did not affect the affinity to both *Torpedo* and neuronal receptors (1.29‐ and 0.7‐fold, respectively).

The importance of the conserved LNTX Phe and Arg residues in high affinity binding to the neuronal α7 nAChRs is further substantiated by the weak inhibitory activities of α‐elapitoxin‐Aa2a of *Acanthophis antarcticus *(Phe and 1st Arg are substituted by Arg29 and Leu33, respectively)^19^ and α‐elapitoxin‐Al2a of *Austrelaps labialis *(only 2nd Arg is replaced by Val36).^20^ Intriguingly, drysdalin lacks the conserved LNTX Phe (replaced by Arg at position 30) and Arg residues (replaced by Leu and Ala at position 34 and 37, respectively), which is three out of the eight functionally conserved residues (see Section 1; Figure 1C). Despite these significant disparities, drysdalin exhibits nanomolar potency to muscle as well as neuronal nAChRs (Table 1). We speculated that reverting mutation of these non‐conserved residues to the functionally conserved residues would enhance the antagonistic activity of drysdalin at the muscle and neuronal nAChRs.

Single (Drys[R30F], [L34R], and [A37R]), double (Drys[R30F,L34R], [R30F,A37R] and [L34R,A37R], and triple (Drys[R30F,L34R,A37R]) mutants (Supporting Information Figure S1) were generated by one‐step site directed plasmid mutagenesis protocol,^35^ and recombinantly expressed in *E coli*, folded and purified by RP‐HPLC (Supporting Information Figures S2, S3). The reduced and refolded proteins showed the mass corresponding to the calculated values (Supporting Information Table S1). The CD spectra of the folded mutants were similar to the full‐length drysdalin (Supporting Information Figure S4), indicating that the mutations did not affect the overall secondary structure of the toxin and presumably the three‐finger fold.

### 3.7 Drysdalin potency at inhibiting ACh‐evoked currents of nAChRs is dependent on non‐conserved residues

**Figure 8:**
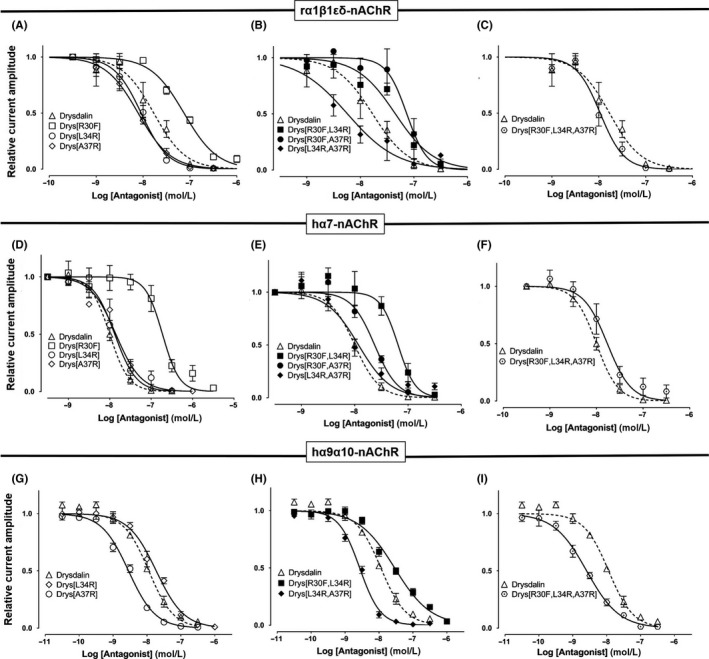
Drysdalin non‐conserved residue mutants inhibition of rα1β1δε, hα7, and hα9α10 nAChRs. Concentration‐response curves of the relative ACh‐evoked current amplitude mediated by rα1β1δε (top), hα7 (middle), and hα9α10 (bottom) nAChRs (n = 3‐7) in the presence of (A, E, H) single mutants Drys[R30F], Drys[L34R], Drys[A37R]; (B, F, I) double mutants Drys[R30F,L34R], Drys[R30F,A37R] and Drys[L34R,A37R], and (C, G, J) triple mutant Drys[R30F,L34R,A37R]. The IC_50 _and n^H^ of all the mutants are listed in Table 3

At the muscle nAChR, Drys[R30F] was ~fourfold less potent (IC_50_ = 74.6 ± 6.7 nmol/L) than drysdalin (IC_50_ = 16.9 ± 3.6 nmol/L) (Figure 8A, Table 3). Aromatic residues (Phe/Trp) at this position are conserved in all LNTXs except for classes 2d, 3a and 3c (Figure 1) and play a crucial role in interacting with Tyr185, Tyr192 of the muscle nAChR through π‐π interactions. In class 2d, 3a and 3c of LNTXs (except 476539402), the conserved Phe/Trp is replaced by Arg resulting in cation‐π interactions between the aromatic nAChR residues and the guanidinium group of Arg.^36^ The reduced potency of Drys[R30F] suggests that cation‐π interaction is preferred over π‐π interaction in the ligand‐binding pocket of the muscle nAChR.

**Table 3:** IC_50_ values of drysdalin and mutants at rodent (r)α1β1δε, human (h)α7 and hα9α10 nAChRs expressed in *Xenopus* oocytes

Antagonist	rα1β1δε			hα7			hα9α10		
	IC_50_ (nmol/L)	n^H^	Fold	IC_50_ (nmol/L)	n^H^	Fold	IC_50_ (nmol/L)	n^H^	Fold
Drysdalin	16.9 ± 3.6	1.3	1.0	10.0 ± 0.6	1.9	1.0	11.3 ± 0.2	1.1	1.0
tDrysdalin	97.6 ± 14	1.4	**5.8**	19.5 ± 2.1	1.2	2.0	Inactive*	–	–
Drys[R30F]	74.6 ± 6.7	1.2	**4.4**	188 ± 23.8	2.1	**18.8**	Inactive*	–	–
Drys[L34R]	9.1 ± 1.1	1.5	0.5	13.2 ± 1.4	1.7	1.3	18.2 ± 1.6	1.1	2.2
Drys[A37R]	7.9 ± 0.8	1.3	0.5	14.1 ± 1.5	1.4	1.4	2.8 ± 0.2	1.1	0.3
Drys[R30F,L34R]	45.1 ± 10.3	1.2	2.7	65 ± 10	2.7	**6.5**	28.0 ± 2.6	0.8	3.4
Drys[R30F,A37R]	73.7 ± 12	2.3	**4.4**	23.6 ± 3.1	2	2.4	Inactive*	–	–
Drys[L34R,A37R]	5.7 ± 1.5	0.9	0.3	15.5 ± 2.4	1.4	1.6	2.6 ± 0.2	1.4	0.3
Drys[R30F,L34R,A37R]	10.7 ± 1.2	1.7	0.6	17.1 ± 2.6	1.7	1.7	2.5 ± 0.2	0.9	0.3

IC_50_ values indicated are with their respective error of the fit (*n* = 3–9).

Fold values indicate the IC_50_ of drysdalin's mutant/IC_50_ of drysdalin on corresponding nAChR subtype, where values in bold indicate >fourfold decrease in IC_50_ of drysalin's mutant compared to drysdalin.

*Indicates concentration tested upto 100 nmol/L.

In contrast, the potency of Drys[L34R] and Drys[A37R] mutants (IC_50_ = 9.1 ± 1.1 nmol/L and 7.9 ± 0.8 nmol/L, respectively) at inhibiting rα1β1δε nAChR was enhanced ~twofold compared to drysdalin (Figure 8A, Table 3), consistent with the fact that positively charged residues at these positions are favourable in the interaction with the muscle nAChR. Similarly, [L34R] mutation improved the potency of Drys[R30F] ~twofold (IC_50_ = 45.1 ± 10.2 nmol/L) although Drys[R30F,A37R] double mutant had comparable potency to the [R30F] mutant (IC_50 _= 73.7 ± 12.0 nmol/L). On the other hand, [L34R, A37R] double mutation with three Arg residues present (R30, R34, and R37), had the highest potency at the muscle nAChR with IC_50_ of 5.7 ± 1.5 nmol/L (Figure 8B, Table 3). Although the triple mutant Drys[R30F,L34R,A37R] was slightly more active (IC_50_ of 10.7 ± 1.2 nmol/L) than drysdalin, its lower activity could be explained by the R30F mutation (Figure 8C, Table 3).

Phe substitution of Arg30 greatly reduced the potency of drysdalin at inhibiting the ACh‐evoked currents of hα7 nAChRs by >18‐fold (IC_50 _= 188.0 ± 23.8 nmol/L) (Figure 8D, Table 3). In contrast, Drys[L34R] (IC_50 _= 13.2 ± 1.4 nmol/L), Drys[A37R] (14.1 ± 1.5 nmol/L), and Drys[L34R, A37R] (IC_50 _= 15.5 ± 2.4 nmol/L) mutants were only slightly less potent than drysdalin at inhibiting ACh‐evoked currents (Figure 8D‐E). Subsequent Arg substitution to residues Leu34 and/or Ala37 of Drys[R30F], resulted in improved activity by ~3‐11‐fold (Drys[R30F,L34R] IC_50 _= 65.0 ± 10.0 nmol/L, Drys[R30F,A37R] IC_50 _= 23.6 ± 3.1 nmol/L (Figure 8E), and Drys[R30F,L34R,A37R] IC_50_ = 17.1 ± 2.6 nmol/L) (Figure 8F). All of the LNTX‐conserved residue substitution reduced the potency of drysdalin at inhibiting hα7 subtype, suggesting distinct contributions of the non‐conserved Arg30, Leu34, and Ala37 residues from other α7‐targeting LNTXs.

Both Drys[R30F] and Drys[R30F,A37R] at 100 nmol/L, did not inhibit hα9α10 ACh‐evoked currents (Table 3). Swapping Leu34 with the positively charged Arg marginally reduced the IC_50_ of drysdalin ~twofold (IC_50 _= 18.2 ± 1.6 nmol/L) whereas, the mutation resulted in functional Drys[R30F] (IC_50 _= 28.0 ± 8.4 nmol/L) although the potency of Drys[R30F,L34R] was ~threefold less than drysdalin (Figure 8H). In contrast, the potency of drysdalin was enhanced ~threefold in Drys[A37R] (IC_50_ = 2.8 ± 0.2 nmol/L), Drys[A37R,L34R] (IC_50_ = 2.6 ± 0.2 nmol/L), and Drys[R30F,A37R,L34R] (IC_50_ = 2.5 ± 0.2 nmol/L) (Figure 8G*‐*I).

### 3.8 Implication on homology and understanding of protein chemistry

Multiple sequence alignment is one of the widely used bioinformatics tool used to determine structure‐function relationships, structural modelling, functional prediction, phylogenetic analysis, and sequence database searching. Structural and functional similarities are predicted based on the identity and similarity between the residues of the proteins where the higher the identity/similarity, the higher the confidence in predictions and conversely, non‐conserved substitutions lead to loss of structure or function. Here we describe for the first time, for drysdalin non‐conserved substitutions continue to retain the functional efficiency of the protein as they maintain similar interactions through altered modes/mechanisms. Our results indicate that further considerations are needed to enhance the functional predictability of proteins using bioinformatics approaches.

## 4 CONCLUSIONS

In LNTXs, eight conserved residues define the binding of the toxins to nAChRs and confer potent postsynaptic neurotoxicity.^15^, ^16^ Uniquely, the conserved one Phe and two Arg residues are absent in drysdalin, replaced by Arg30 and, Leu34 and Ala37, respectively. It selectively antagonizes rodent muscle (α1)_2_β1δε, and human α7 and α9α10 nAChRs. Replacing Leu34 and Ala37 residues with the conserved LNTX Arg residue had minimal impact on the potency at the nAChRs. In contrast, substituting Arg30 residue with Phe impaired the inhibitory activity of drysdalin. In addition to the residues in loop II, we found that the C‐terminal tail plays a critical role in recognizing α9α10 nAChR. The C‐terminal tail also affects the reversibility of drysdalin at the muscle and α7 nAChRs. Thus, although the conservation of functional residues in a protein family is important in retaining its function, it may not be absolute.

## 5 CONFLICT OF INTEREST

The authors declare that they have no conflict of interest.

## AUTHORS CONTRIBUTIONS

R.C. performed the mutagenesis, protein purification and pharmacology experiments and their analysis. H.S.T. performed the electrophysiology experiments and analysed the data. V.A.L.S. and S.C. performed the initial electrophysiology and pharmacology experiments, respectively. R.C., D.J.A., and R.M.K. conceptualized the project and wrote the manuscript. All authors read the manuscript and were engaged in the preparation of the final form.
