# Supplementary figures and images for: Drysdalin, an antagonist of nicotinic acetylcholine receptors highlights the importance of functional rather than structural conservation of amino acid residues
Source: FASEB Bioadv. 2019 Jan 10;1(2):115–31. doi: 10.1096/fba.1027 (PMC6996315; doi:10.1096/fba.1027)

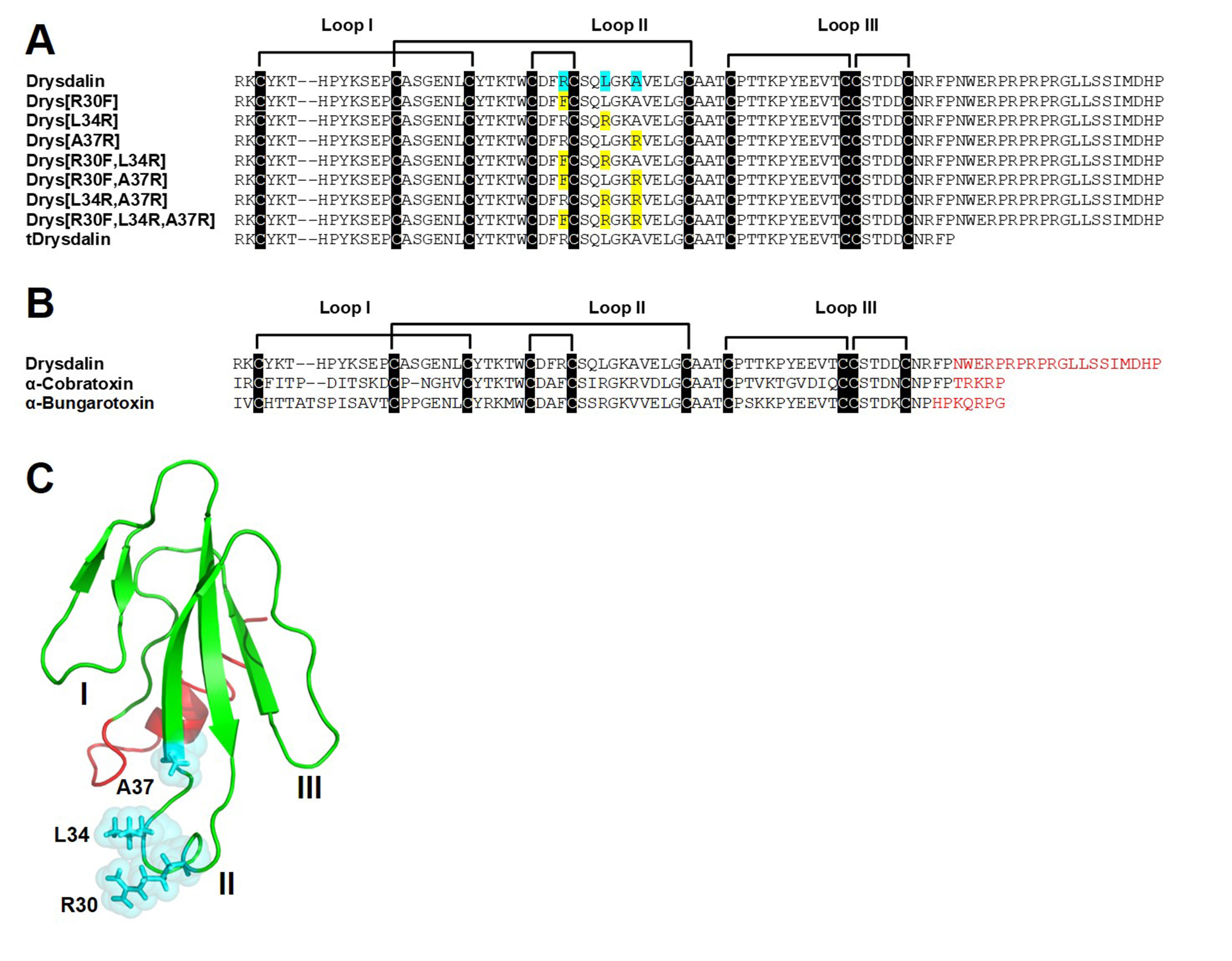

Supplement: Supplementary file 1 [file FBA2-1-115-s001.jpg]

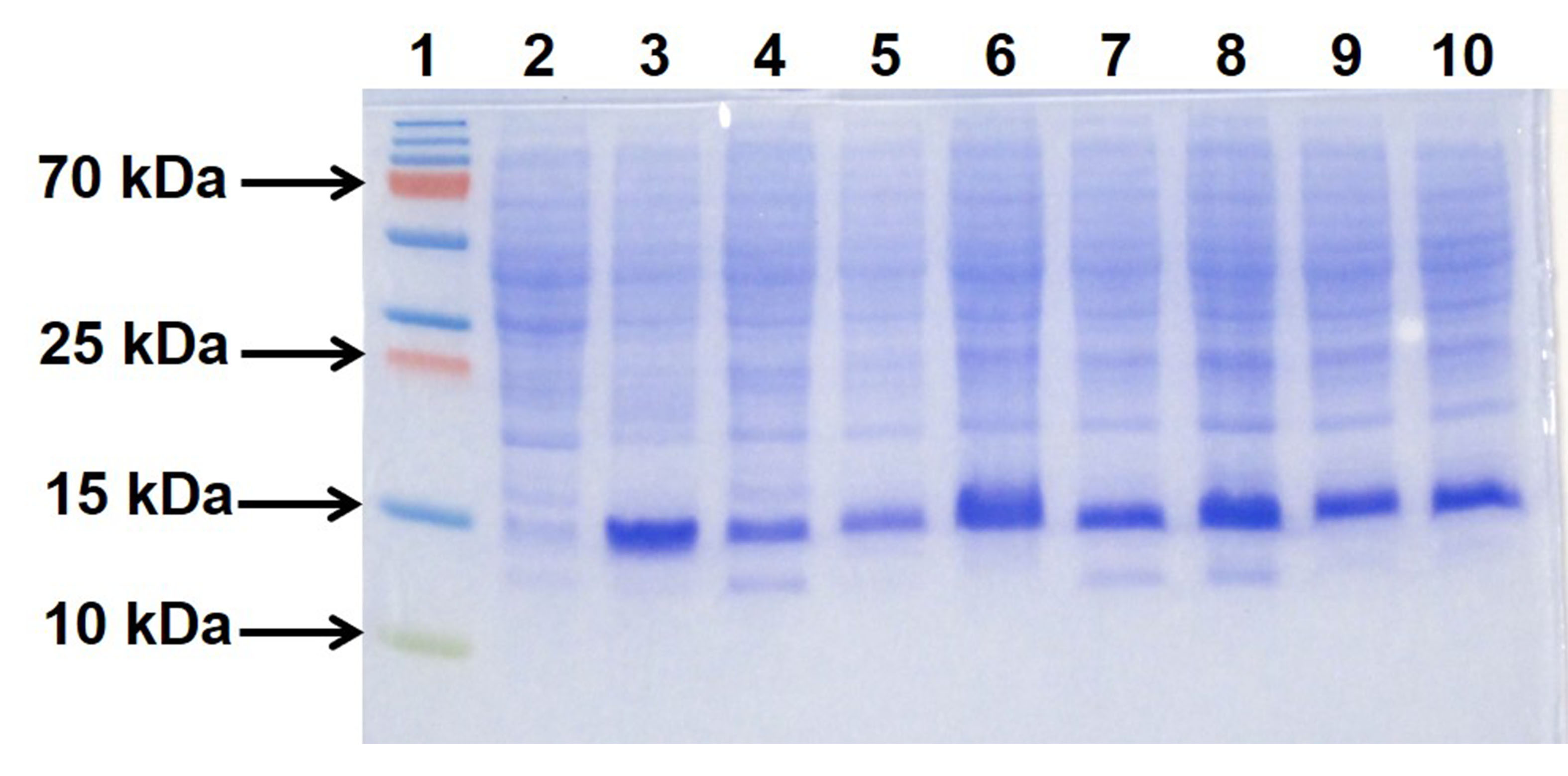

Supplement: Supplementary file 2 [file FBA2-1-115-s002.jpg]

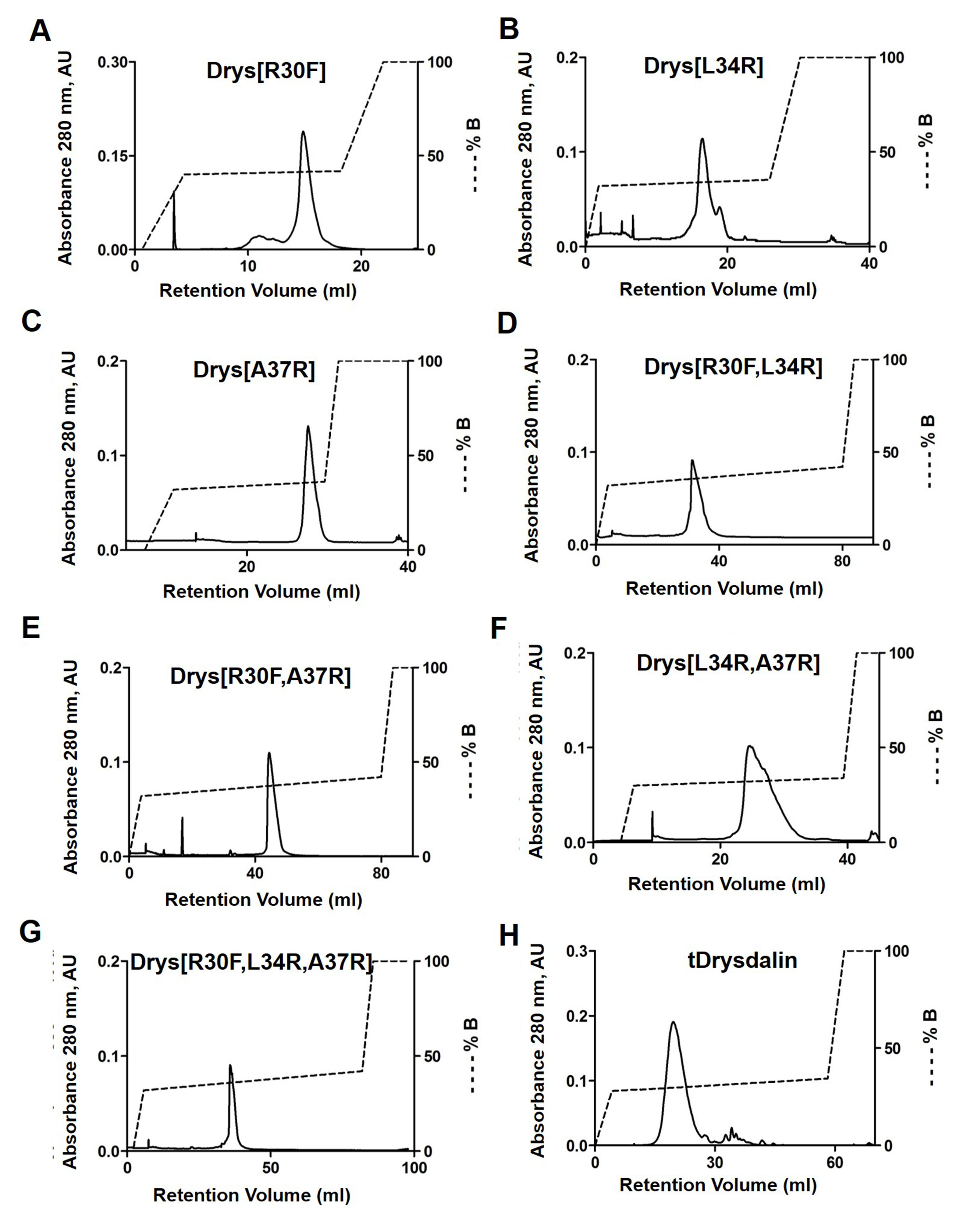

Supplement: Supplementary file 3 [file FBA2-1-115-s003.jpg]

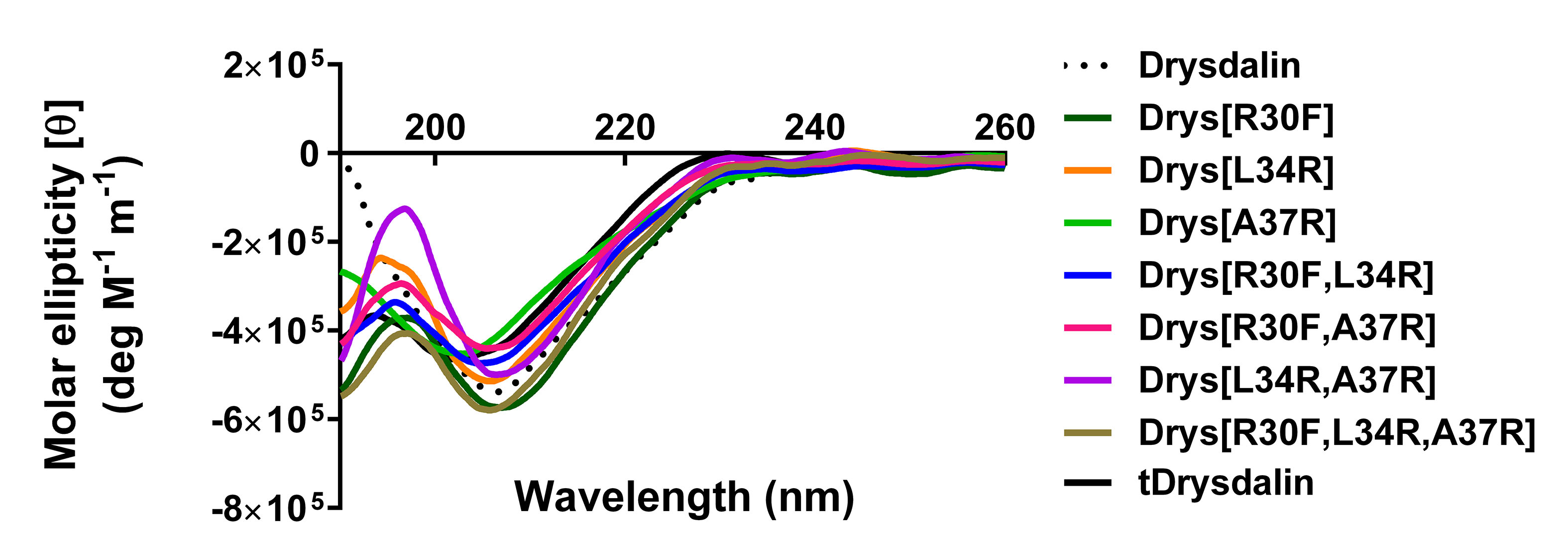

Supplement: Supplementary file 4 [file FBA2-1-115-s004.jpg]

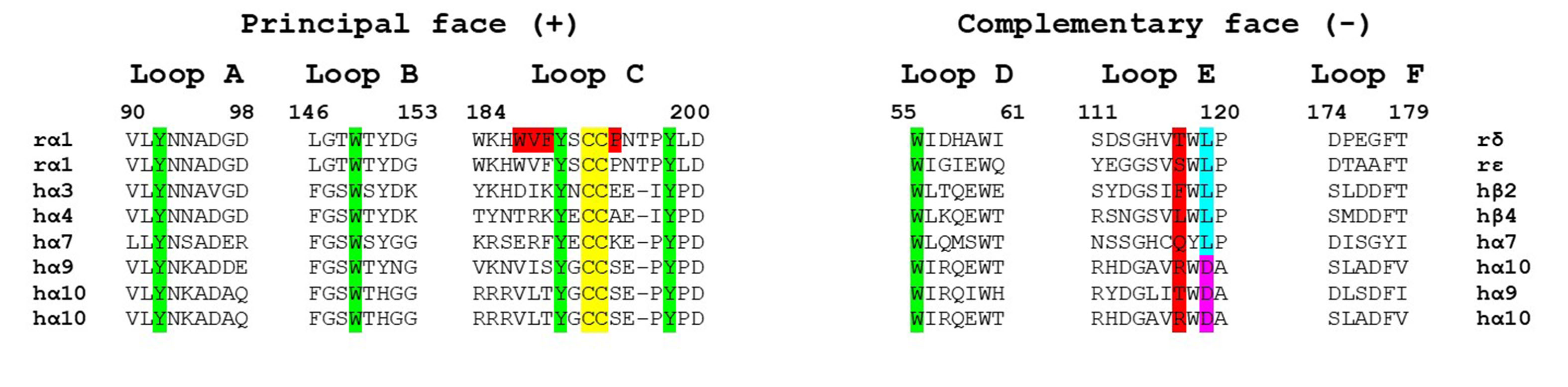

Supplement: Supplementary file 5 [file FBA2-1-115-s005.jpg]

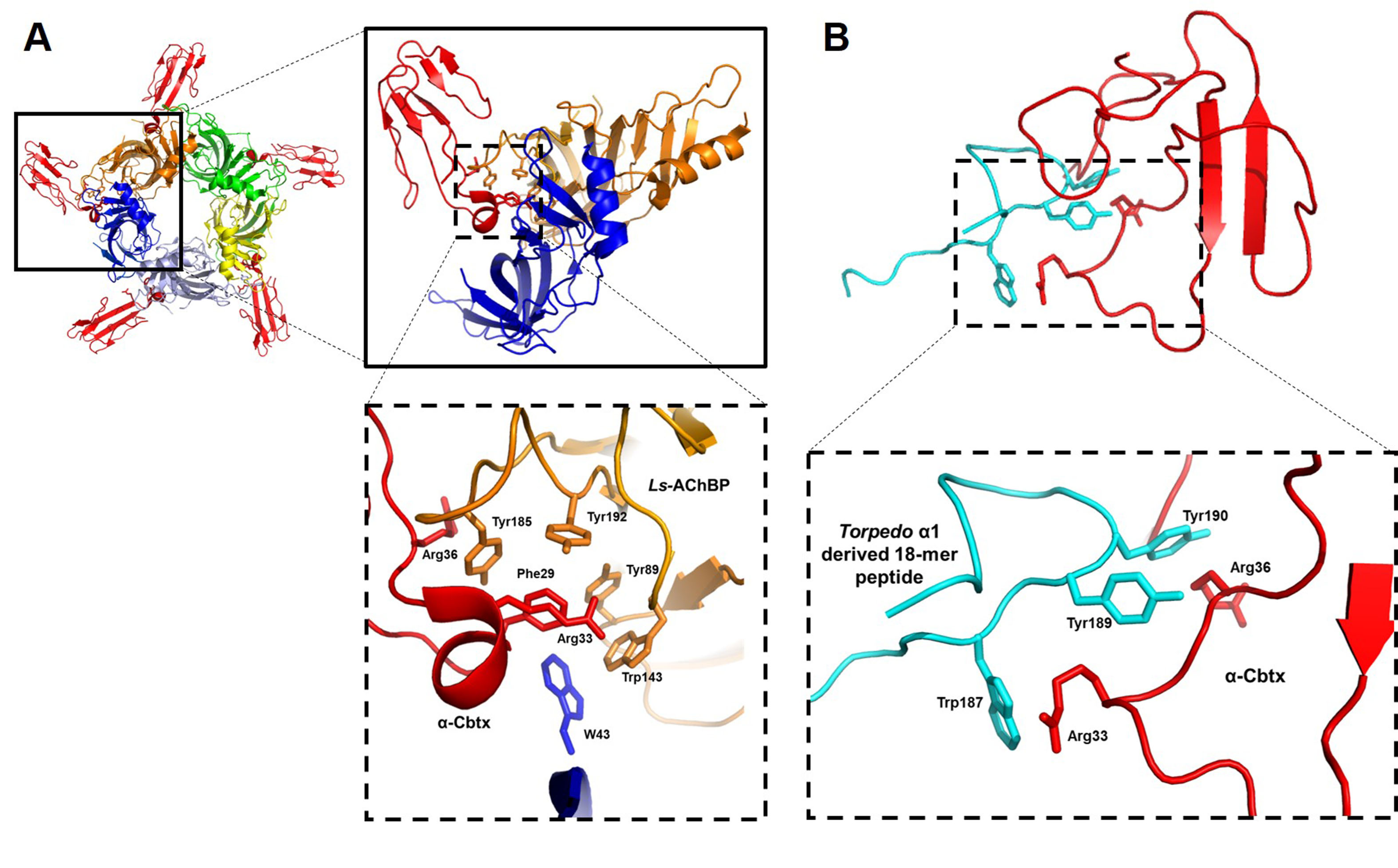

Supplement: Supplementary file 6 [file FBA2-1-115-s006.jpg]

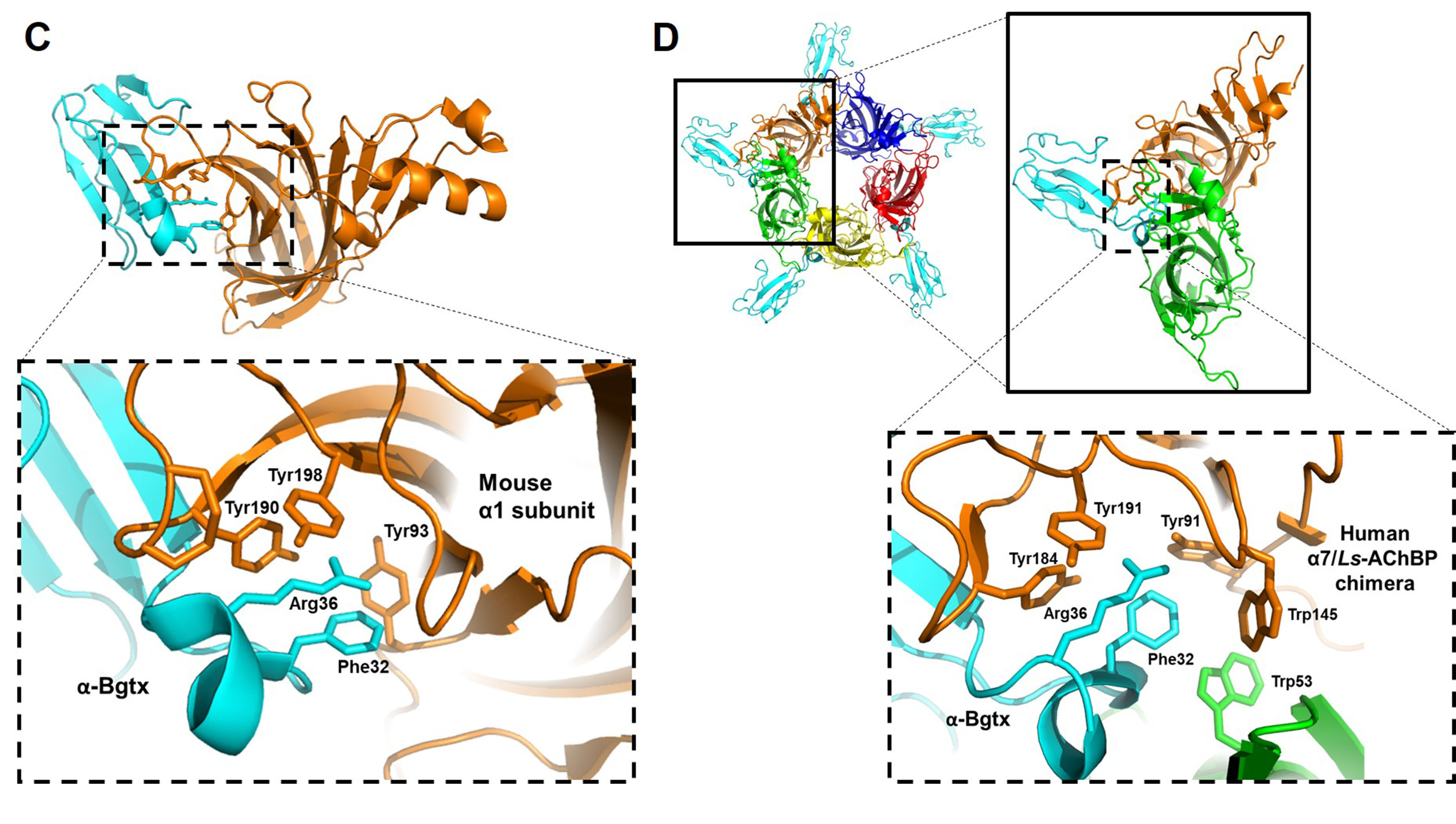

Supplement: Supplementary file 7 [file FBA2-1-115-s007.jpg]
