# Supplementary material for: Drysdalin, an antagonist of nicotinic acetylcholine receptors highlights the importance of functional rather than structural conservation of amino acid residues
Source: FASEB Bioadv. 2019 Jan 10;1(2):115–31. doi: 10.1096/fba.1027 (PMC6996315; doi:10.1096/fba.1027)
Supplement: Supplementary file 8 [file FBA2-1-115-s008.docx]

**Supplementary Information**

**Drysdalin, a long-chain neurotoxin from the venom of *Drysdalia coronoides*, highlights the importance of functional rather than structural conservation in its activity at nicotinic acetylcholine receptors**

Ritu Chandna^1#^, Han-Shen Tae^2#^, Victoria A. L. Seymour^2^, Shifali Chathrath^1^, David J. Adams^2^ and R Manjunatha Kini^1*^

^1^ Protein Science Laboratory, Department of Biological Sciences, National University of Singapore, Singapore 117543

^2^ Illawarra Health and Medical Research Institute (IHMRI), University of Wollongong, Wollongong, NSW 2522, Australia

^3^ Burnet Institute, Melbourne, VIC 3004, Australia

# Equal first authors

**EXPERIMENTAL PROCEDURES**

***Materials***

The DNA fragment encoding drysdalin with codon optimization for overexpression in *Escherichia coli* was synthesized by GenScript Corporation (Piscataway, NJ, USA). High Fidelity PCR kit from Kapa Biosystems (Wilmington, MA, USA) and DNA ladders from Invitrogen (Grand Island, NY, USA) were used. Custom oligonucleotides were purchased from 1st Base Pte. Ltd., Singapore. *E. coli* SHuffle® T7 Express competent cells were obtained from New England Biolabs (Beverly, MA, USA). Fast digest restriction endonucleases was purchased from Fermentas fast digest (Ontario, Canada). Precision Plus Protein Dual-Color Standards (SDS/PAGE markers) were purchased from Bio-Rad Laboratories (Hercules, CA, USA). The RP-Jupiter C18 [5 *μ*, 300 Å (1 Å=0.1 nm), 4.6 mm×250 mm] column was purchased from Phenomenex. Acetonitrile (ACN) was purchased from TEDIA® (Fairfield, OH, USA) and trifluoroacetic acid (TFA) from Merck KGaA (Darmstadt, Germany). All chemicals and reagents used were of analytical grade. The drugs used in organ bath experiments, acetylcholine (ACh) and carbachol (CCh), were purchased from Sigma Aldrich (St. Louis, MO, USA) and, Bgtx and Cbtx were purchased from Latoxan (Valence, France).

***Animals***

Swiss albino mice were acquired from the National University of Singapore Laboratory Animal Center and acclimatized to the Animal Holding Unit for at least 3 days before the experiments. Four mice were housed per cage at 23°C and 60% relative humidity with 12 h light/dark cycle, light at 7 am with food and water available *ad libitum*) Domestic chicks (*Gallus gallus domesticus*) were purchased from Chew’s Agricultural Farm, Singapore and delivered on the day of experimentation. Animals were euthanized with 100% CO_2_. All experiments were conducted according to the Protocol (103/08A) approved by the National University of Singapore Institutional Animal Care and Use Committee.

***Sub-cloning of drysdalin***

The 261 bp DNA fragment of the synthetic drysdalin gene in pUC17 was amplified by PCR using the forward primer 5’-GGGGATCCCGTAAATGCCTATAAAACC-3’and the reverse primer 5’-CAGCGGCCGCTCACGGATGATC-3’ with BamH1 and Not1 restriction sites, respectively followed by sub-cloning into pET-M (a modified pET-32a vector).

***Site directed mutagenesis***

The PCR mix (50 μl) contained 1 μl (< 20 ng) of template plasmid, 0.2 mM dNTPs, 0.2 μM of both primers, HiFi buffer at 1 X final concentration and 1 μl of polymerase. Three-step thermal cycling involved: 95°C/3 min followed by 25 cycles of 98°C/20 s (denaturation), T°C/20 s (annealing), 72°C/4 min (extension) and a final extension of 72°C/5 min followed with DpnI digestion at 37°C for 15 min and transformed into JM109 cells. Mutation was confirmed by DNA sequencing. Mutagenesis primers along with the annealing temperatures (T) and template plasmid are listed in Table S1.

***Recombinant protein expression***

The recombinant plasmids were transformed in *E. coli* SHuffle® competent cells and cultured in Luria Bertani medium supplemented with ampicillin (100 μg/ml). Protein expression was induced at an A_600_ of 0.6 with 1 mM IPTG and harvested after 4 h at 37°C. Bacterial cells were resuspended in lysis buffer (50 mM Tris-HCl, 150 mM NaCl, 1 mM EDTA and 0.1% Triton X-100, pH 8.0), sonicated for lysis. The pellet obtained after centrifugation was washed twice with buffer containing 1% Triton X-100 and finally resuspended in denaturing buffer (50 mM Tris-HCl, 150 mM NaCl, 6 M guanidine hydrochloride, pH 8.0). Expression of the recombinant protein was analyzed by SDS/PAGE on a 15% Tris-Tricine gel.

***Protein purification and refolding***

The solubilized pellet was reduced with 100 mM DTT for 2 h at 37°C followed by centrifugation. Reduced protein was filtered through a 0.45 μm filter and purified to homogeneity using RP-HPLC on a Jupiter C18 column (4.5 mm × 2.1 mm) with solvent A (0.1% TFA) and solvent B (80% ACN in 0.1% TFA) on an AKTA^TM^ system (GE Healthcare Life Sciences, Singapore). The molecular mass and homogeneity were evaluated by ESI-MS using a LCQ Fleet^TM^ Ion Trap Mass Spectrometer (Thermo Scientific, Waltham, MA, USA) in positive ion mode. ACCELA-600 pump was used for solvent delivery (50% ACN in 0.1% formic acid) at 100 μl/min. Full scan data were acquired over the range 800 to 2000 m/z, with 0.1 Da step mass and analysed using ProMass Deconvolution™ software.

Fractions with the expected molecular mass were pooled, lyophilized and dissolved in buffer containing 3 M guanidine hydrochloride, reduced with 50 mM DTT and diluted drop-wise into the refolding buffer (50 mM Tris-HCl, 1 mM EDTA, 0.5 M guanidine hydrochloride, 20% glycerol, 1 mM reduced glutathione, 1 mM oxidized glutathione, pH 8.0) such that the final protein concentration is 0.05 mg/ml. Refolding was carried out at 4°C for 4 days with constant stirring at 50 rpm and stopped by acidification with TFA. The refolded protein was purified by RP-HPLC and the molecular mass was determined by ESI-MS.

***CD spectroscopy***

Far-UV CD spectra (260–190 nm) were recorded using a Jasco J-810 spectropolarimeter as described previously (1). Protein sample (10-20 μM) was dissolved in MilliQ water and placed in a 0.1 cm path length cuvette. Measurement was done at 50 nm/min under nitrogen atmosphere at 0.1 nm data pitch and 1 nm bandwidth. The average of three scans was taken to increase the signal-to-noise ratio.

***In vivo toxicity test***

Drysdalin (200 μl dissolved in 20 mM phosphate buffer saline (PBS) pH=7.4) was injected intraperitoneally using a 27-gauge 0.5-inch needle (BD Biosciences, New Jersey) into male Swiss albino mice at doses of 0.25 and 3 mg/kg (*n*=1). The symptoms of envenomation were observed, and in the event of death, the time of death was noted. The control group was injected with 200 μl of PBS (n=2) and after 1 h, all surviving mice were euthanized by CO_2_ overdose. Post-mortem examinations were conducted on all animals.

***Bioinformatics***

Multiple sequence alignment of LNTXs was done using the Clustal Omega. MEME (Mutiple Em for Motif Elicitation) diagrams were generated using MEME (Version 4.12.0) using the online server (http://meme-suite.org/tools/meme). Numbers on the X-axis indicate residue position in the protein sequence (including the spaces).

***Ex vivo chick biventer cervicis muscle (CBCM) organ bath***

The CBCM nerve-skeletal muscle was isolated from 1-4 days old chicks and mounted in a 6 ml-organ bath chamber containing carbogen (5% CO_2_ in O_2_)-aerated Krebs solution consisting of (in mM) 118 NaCl, 4.8 KCl, 1.2 KH_2_PO_4_, 2.5 CaCl_2_, 25 NaHCO_3_, 2.4 MgSO_4_, and 11 D-(+) glucose); pH 7.4, at 37°C as described previously (2). The resting tension of the tissues was maintained at 1–2 g, and preparations were allowed to equilibrate for 30–45 min. Electrical field stimulation was applied through platinum ring electrodes connected to a Grass stimulator S88 (Grass Instruments, West Warwick, RI, USA) and the contractile response magnitude was measured in gram tension. Data were continuously recorded on PowerLab/Chart 5 data acquisition system via a force displacement transducer (Model MLT0201, AD Instruments, Bella Vista, NSW, Australia). Neuromuscular blockade by drysdalin is expressed as percentage of the twitch height in the absence of drysdalin to the twitch height 30 min post exposure to drysdalin. The half-maximal inhibitory concentration (IC_50_) was determined from concentration-response curve fitted to a non-linear regression function and reported with error of the fit. Recovery from complete neuromuscular blockade by drysdalin was assessed by washing out the toxin with Krebs solution at 30 min intervals (three cycles of 30 s on pulse, 30 s off pulse) over a 120-min period after 80% blockade of the twitch responses.

***Electrophysiology***

RNA preparation, oocyte preparation and expression of nAChRs were performed as described previously (3). Briefly, plasmid DNAs encoding rat (α1, β1, and δ), mouse (ε) and human (α3, α4, α7, α9, α10, β2 and β4) nAChR subunits were linearized with appropriate restriction enzymes (NEB, Ipswich, MA, USA) and cRNA was synthesised using SP6 or T7 in vitro transcription kit (mMessage mMachine, Ambion, Foster City, CA, USA).Stage V-VI oocytes were obtained from *Xenopus laevis*, defolliculated with 1.5 mg/ml Type II collagenase (Worthington Biochemical Corp., Lakewood, NJ, USA) in OR-2 solution (82.5 mM NaCl, 2 mM KCl, 1 mM MgCl_2_ and 5 mM HEPES, pH 7.4). Oocytes were injected with 5 ng of cRNA (35 ng for human α9α10) using glass pipettes pulled from glass capillaries. Oocytes were incubated at 18ºC in sterile ND96 solution (96 mM NaCl, 2 mM KCl, 1 mM CaCl_2_, 1 mM MgCl_2_ and 5 mM HEPES, pH 7.4) supplemented with 5% FBS, 100 μg/l gentamycin and 100 units/ml penicillin-streptomycin. All protocols were approved by the University of Sydney Animal Ethics Committee. Electrophysiological recordings were carried out 2–7 days after microinjection. Two-electrode voltage clamp recordings from oocytes were carried out at room temperature (20-23ºC) using a GeneClamp 500B amplifier (Molecular Devices Corp., Sunnyvale, CA, USA) at a holding potential −80 mV. Voltage-recording and current-injecting electrodes were pulled from borosilicate glass (GC150T-7.5, Harvard Apparatus Ltd., Holliston, MA, USA) and had resistances of 0.3–1 MΩ when filled with 3 M KCl.

Oocytes expressing human α9α10 nAChR were incubated with 100 μM BAPTA-AM (Sigma-Aldrich, St. Louis, MO, USA) at 18 °C for ~3 h before recording and perfused with ND115 solution containing (in mM): 115 NaCl, 2.5 KCl, 1.8 CaCl2, and 10 HEPES at pH 7.4, whereas other nAChR-expressing oocytes were perfused with ND96 solution. All oocytes were perfused at 2mL/min using a continuous push/pull syringe pump system. nAChR-mediated currents were evoked by applying acetylcholine (ACh) at a half-maximal effective concentration (EC_50_) of each subtype followed by washouts of 180s between ACh applications. Oocytes were incubated with the toxin for 5 min before ACh was co-applied. All toxin solutions were made in ND96/ND115 containing 0.1 % bovine serum albumin. Peak ACh-evoked current amplitude before and after toxin incubation was recorded using pClamp 9 and measured using Clampfit 10.7 (Molecular Devices, Sunnyvale, CA, USA).

*Data analysis*

Concentration-response curves were obtained by plotting averaged relative peak current amplitude values (I/I_control_) against toxin concentration and fitted to a the Hill equation (I = I_control_{[TX]^n^/(IC_50_^n^ + [TX]^n^)}, where I_control_ = maximum peak current amplitude, [TX] = toxin concentration, n = Hill coefficient, and IC_50_ = half-maximal inhibitory concentration ). All data were pooled (n = 4 to 10) and represents mean ± standard error of the mean (SEM). Differences were deemed statistically significant when p < 0.05. Computation was done using GraphPad Prism 6.03 (GraphPad Software, Inc., La Jolla, CA, USA).

**Figure legends**

**Figure S1: Drysdalin mutants**

(A) Amino acid sequences of drysdalin and mutants. The five disulphide bridges (bold black lines) are formed between the cysteine residues (black columns). Mutated residues of loop II are highlighted in yellow. (B) Amino acid sequences of drysdalin, α-cobratoxin and α-bungarotoxin. The truncated residues are highlighted in red. (C) 3D-structure of drysdalin was modelled by I-TASSER (<https://zhanglab.ccmb.med.umich.edu/I-TASSER/>) (4). Truncated C-terminus (in red) and side-chains of the amino acid residues that were mutated are shown.

**Figure S2: Recombinant expression of drysdalin mutants**

SDS-PAGE analysis of recombinant expression of drysdalin mutants. Lane 1: protein ladder; lane 2: whole-cell lysate before protein induction; lanes 3 to 8: whole-cell lysates of tDrysdalin, Drys[R30F], Drys[L34R], Drys[A37R], Drys[R30F,L34R], Drys[R30F,A37R], Drys[L34R,A37R] and Drys[R30F, L34R, A37R], respectively, after protein induction.

**Figure S3:** **Purification of refolded drysdalin mutants**

HPLC profiles of refolded (A) Drys[R30F], (B) [Drys[L34R], (C) Drys[A37R], (D) Drys[R30F,L34R], (E) Drys[R30F,A37R], (F) Drys[L34R,A37R] and (G) Drys[R30F, L34R, A37R]. Refolded mutants were eluted from the column at a flow rate of 1 ml/min with gradient of 32-42% buffer B over 20 column volume on a Jupiter C18 (5 μ, 300 Å, 4.5 x 4.6 mm) analytical column.

**Figure S4: CD spectra of drysdalin mutants**

Far UV CD spectra of refolded drysdalin and mutants. The proteins were dissolved in MilliQ water (10-20 μM) and the CD spectra recorded using a 0.1 cm path-length cuvette.

**Figure S5: Functional loops of various subunits of muscle and neuronal nAChRs**

Functional loops were identified from previously published data.(5)^,^(6)^,^(7) In the muscle nAChR, loops A, B and C (principal binding face) are contributed by the α1 subunit and, loops D, E and F (complementary binding face) are contributed by the δ or ε subunit. In the neuronal nAChRs, loops A, B and C are from either the α3, α4, α7, α9 or α10 subunits and loops D, E and F are from either the α7, α9, α10, β2 or β4 subunit. The residues as numbered according to the rat α1 subunit. The conserved aromatic residues that form the ‘aromatic cage’ and cysteine residues in the Cys-loop are highlighted in green and yellow, respectively. Residues in the binding site that are different in the various subunits are highlighted in red, cyan and magenta.

**Figure S6: Interactions between LNTXs and nAChRs or their surrogates**

(A) α-Cbtx (red) bound to the principal (orange) and complementary (blue) faces of *Ls*-AChBP (PDB ID: 1YI5). Magnification of the α-Cbtx binding site (dotted box) where π-π interactions between α-Cbtx Phe29 and Ls-AChBP Tyr185 and Tyr192, and cation-π interactions between Tyr185 and α-Cbtx Arg33 and Arg36 are involved. (B) Solution structure of α-Cbtx (red) in complex with α1 cognate peptide (cyan) (PDB ID: 1LXH). Magnification of the α-Cbtx binding site (dotted box) where α-Cbtx Arg33/36 and *Torpedo*-α1 cognate peptide residues Trp187, Tyr189 and Tyr190 for cation-π interactions. (C) α-Bgtx (cyan) bound to the mouse α1 extracellular domain (orange) (PDB ID: 2QC1). Magnification of the α-Bgtx binding site (dotted box) where cation-π interactions between α-Bgtx Phe32 and Arg36 with mouse α1 Trp93, Tyr190 and Tyr198 are involved. (D) α-Bgtx (cyan) bound to an interface of the α7/*Ls*-AChBP chimaera (orange and green) (PDB ID: 4HQP). Magnification of the α-Bgtx binding site (dotted box) where cation -π interactions are formed between α-Bgtx Phe32 and Arg36 with α7 Tyr184. In addition, α-Bgtx Arg36 interacts with α7 residues Tyr91, Trp145 and Tyr191 via the same interactions.

**SUPPLEMENTARY FIGURES:**

**Figure S1:**


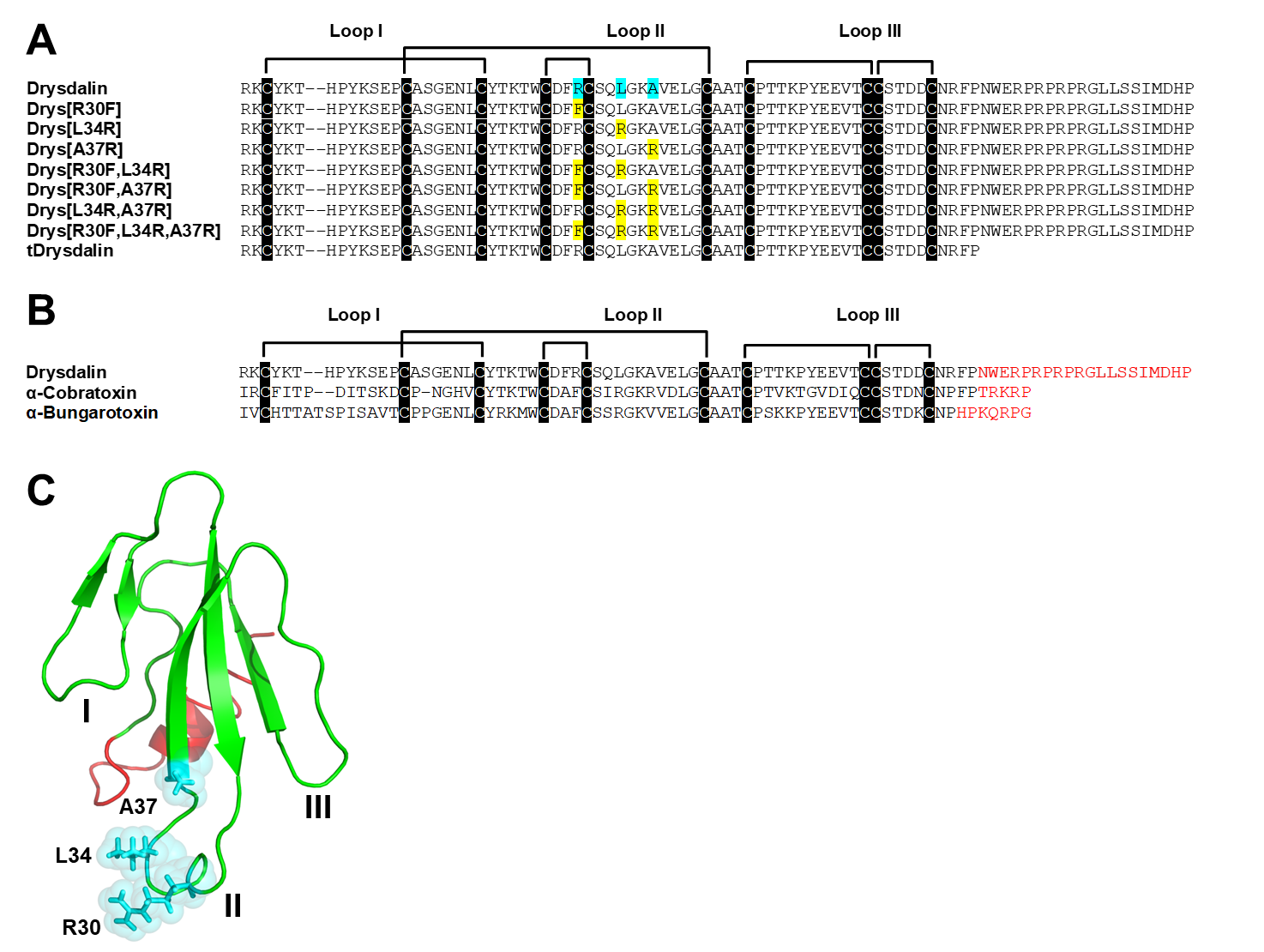


**Figure S2:**

**
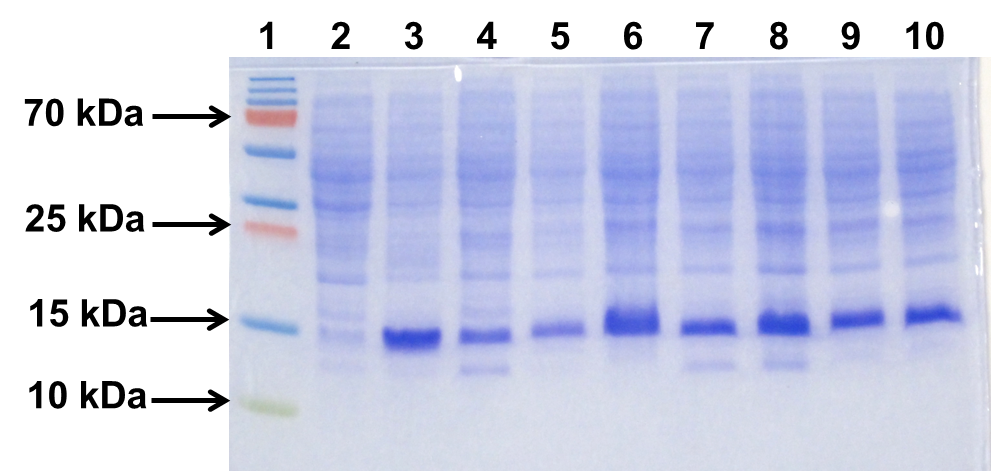
**

**Figure S3:**


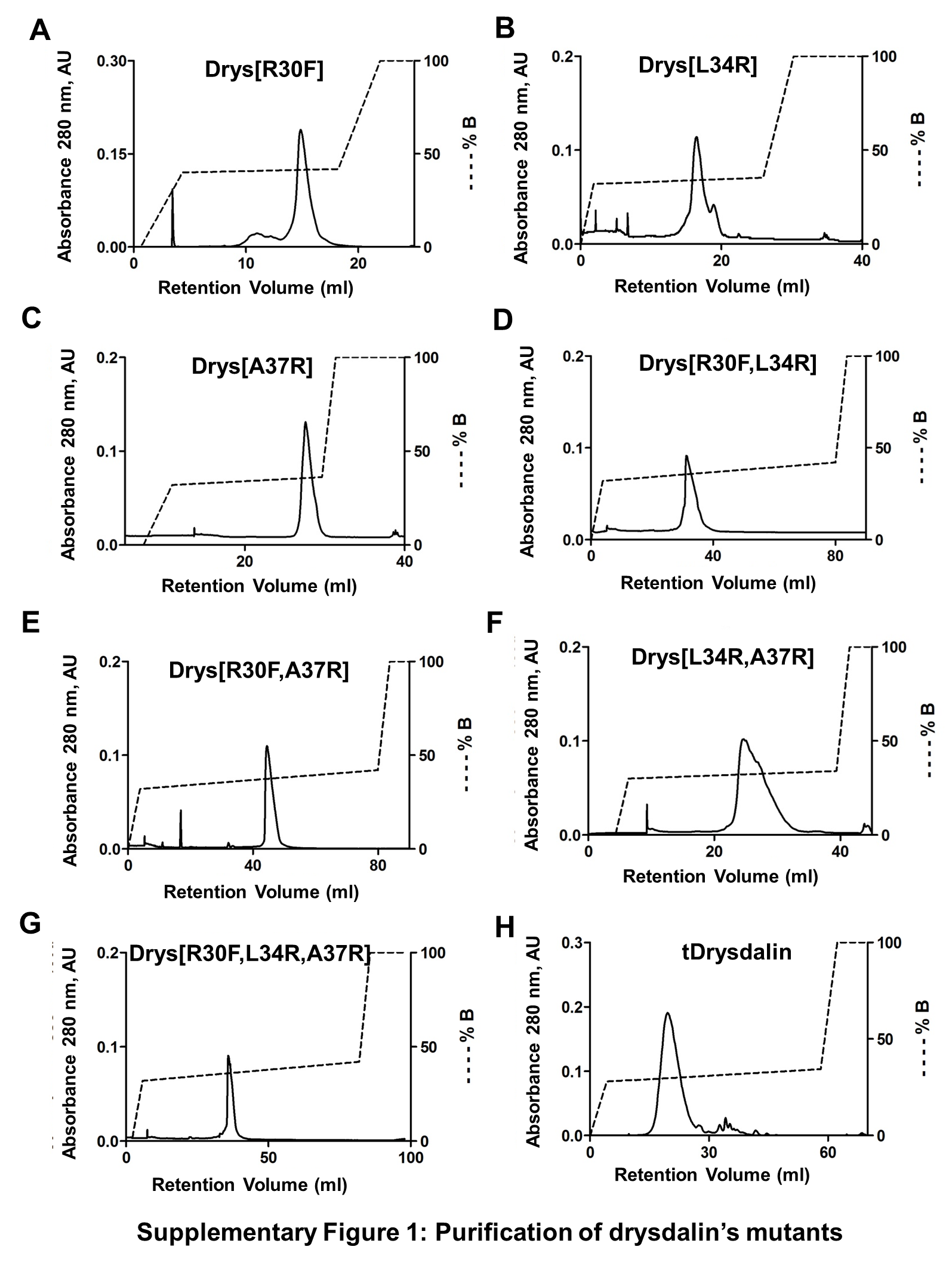


**Figure S4:**

**
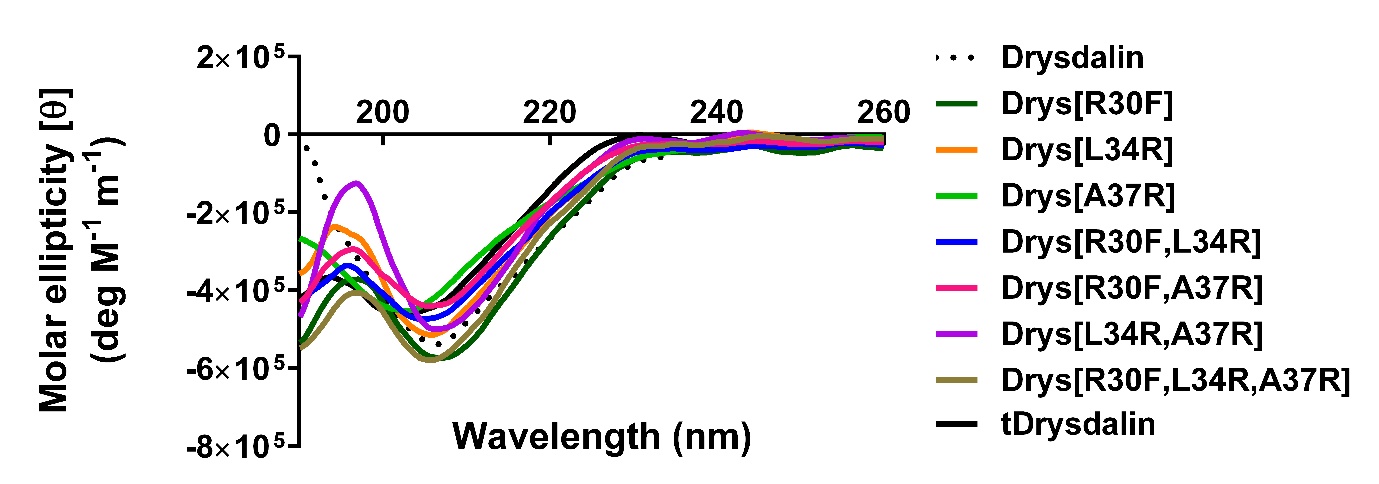
**

**Figure S5:**

|  | **Principal face (+)** | | |  | **Complementary face (-)** | | |  |
| --- | --- | --- | --- | --- | --- | --- | --- | --- |
|  | **Loop A** | **Loop B** | **Loop C** |  | **Loop D** | **Loop E** | **Loop F** |  |
|  | **90 98** | **146 153** | **184 200** |  | **55 61** | **111 120** | **174 179** |  |
| **rα1** | VLYNNADGD | LGTWTYDG | WKHWVFYSCCPNTPYLD |  | WIDHAWI | SDSGHVTWLP | DPEGFT | **rδ** |
| **rα1** | VLYNNADGD | LGTWTYDG | WKHWVFYSCCPNTPYLD |  | WIGIEWQ | YEGGSVSWLP | DTAAFT | **rε** |
| **hα3** | VLYNNAVGD | FGSWSYDK | YKHDIKYNCCEE-IYPD |  | WLTQEWE | SYDGSIFWLP | SLDDFT | **hβ2** |
| **hα4** | VLYNNADGD | FGSWTYDK | TYNTRKYECCAE-IYPD |  | WLKQEWT | RSNGSVLWLP | SMDDFT | **hβ4** |
| **hα7** | LLYNSADER | FGSWSYGG | KRSERFYECCKE-PYPD |  | WLQMSWT | NSSGHCQYLP | DISGYI | **hα7** |
| **hα9** | VLYNKADDE | FGSWTYNG | VKNVISYGCCSE-PYPD |  | WIRQEWT | RHDGAVRWDA | SLADFV | **hα10** |
| **hα10** | VLYNKADAQ | FGSWTHGG | RRRVLTYGCCSE-PYPD |  | WIRQIWH | RYDGLITWDA | DLSDFI | **hα9** |
| **hα10** | VLYNKADAQ | FGSWTHGG | RRRVLTYGCCSE-PYPD |  | WIRQEWT | RHDGAVRWDA | SLADFV | **hα10** |

**Figure S6:**

**B**

**A**

**Figure S6:**

**D**

**C**


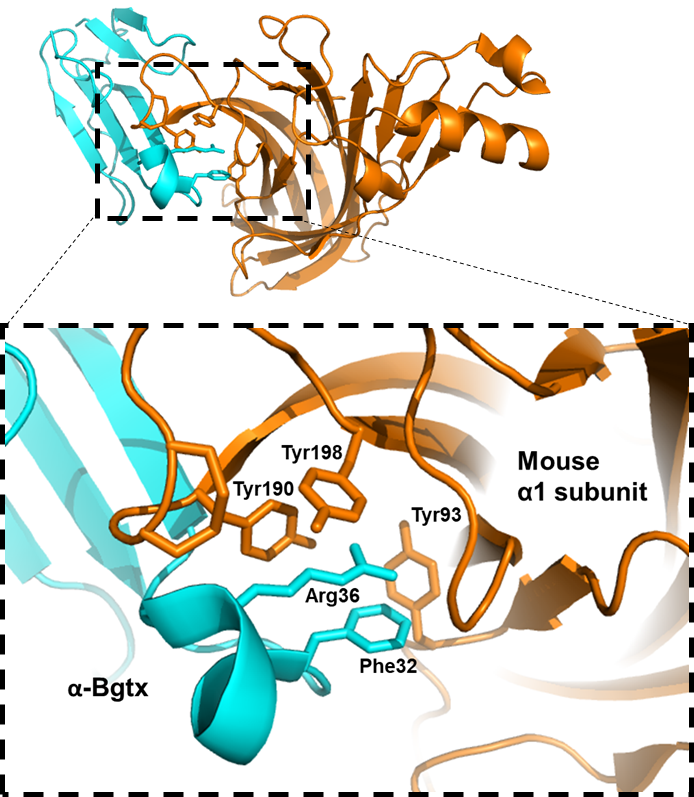

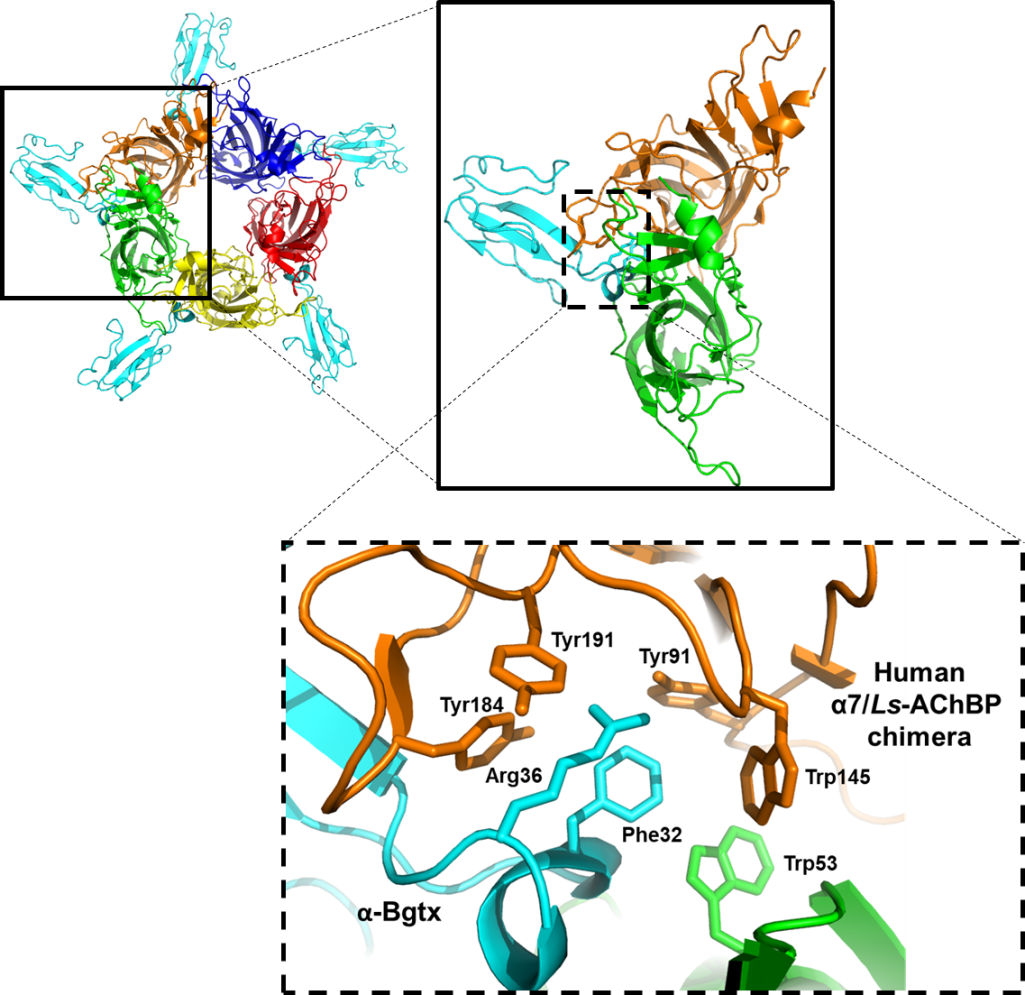


**SUPPLEMENTARY TABLES:**

**Table S1: MS data calculated and measured for drysdalin and mutants**

Table S2: Primers for site directed mutagenesis for generating drysdalin’s mutant plasmids


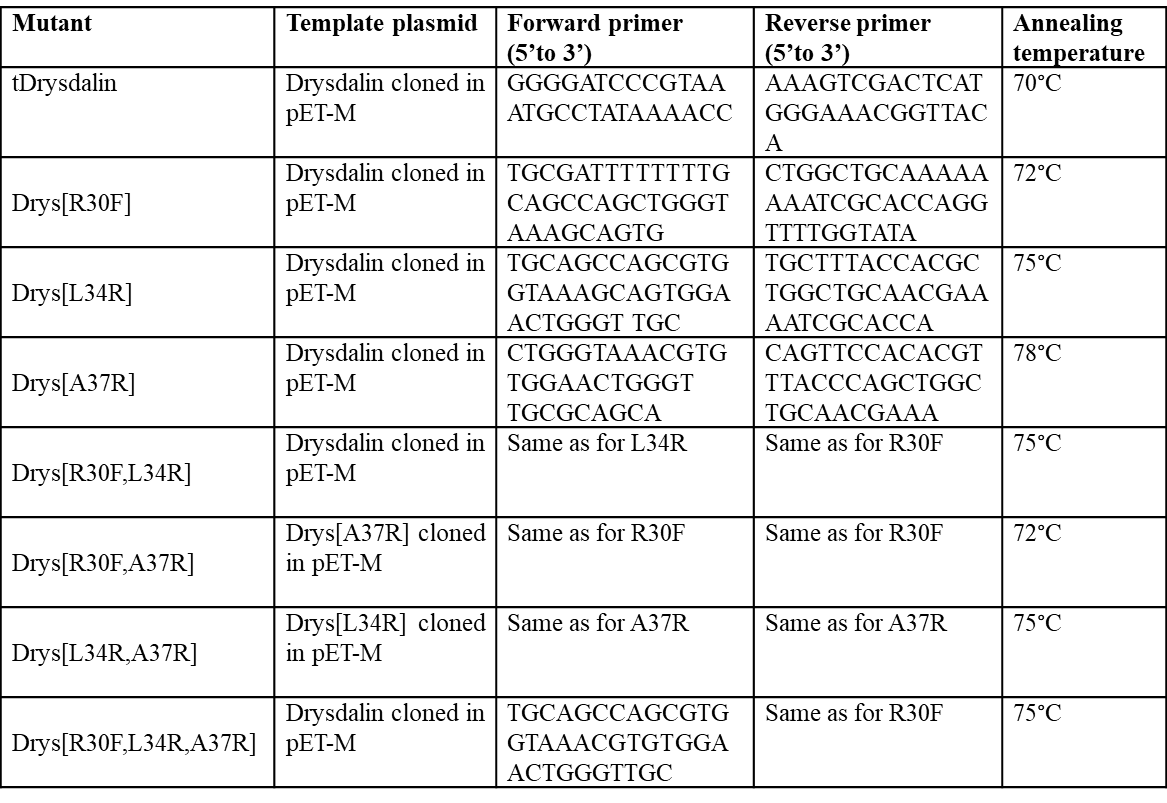


**References:**

1. Rajagopalan, N., Pung, Y. F., Zhu, Y. Z., Wong, P. T., Kumar, P. P., and Kini, R. M. (2007) Beta-cardiotoxin: a new three-finger toxin from *Ophiophagus hannah* (king cobra) venom with beta-blocker activity. *FASEB Journal* **21**, 3685-3695

2. Ginsborg, B. L., and Warriner, J. (1960) The isolated chick biventer cervicis nerve-muscle preparation. *British Journal of Pharmacology and Chemotherapy* **15**, 410-411

3. Bertrand, D., Cooper, E., Valera, S., Rungger, D., and Ballivet, M. (1991) Electrophysiology of neuronal nicotinic acetylcholine receptors expressed in Xenopus oocytes following nuclear injection of genes or cDNAs. Electrophysiology and Microinjection in *Methods in Neurosciences* Edited by Conn, P. M., Vol. **4**, 174-193

4. Roy, A., Kucukural, A., and Zhang, Y. (2010) I-TASSER: a unified platform for automated protein structure and function prediction. *Nature protocols* **5**, 725-738

5. Grutter, T., and Changeux, J.-P. Nicotinic receptors in wonderland. *Trends in Biochemical Sciences* **26**, 459-463

6. Karlin, A. (2002) Emerging structure of the nicotinic acetylcholine receptors. *Nature Reviews. Neuroscience* **3**, 102-114

7. Fruchart-Gaillard, C., Gilquin, B., Antil-Delbeke, S., Le Novère, N., Tamiya, T., Corringer, P.-J., Changeux, J.-P., Ménez, A., and Servent, D. (2002) Experimentally based model of a complex between a snake toxin and the α7 nicotinic receptor. *Proceedings of the National Academy of Sciences USA* **99**, 3216-3221
